# ORAL PRESENTATIONS

**DOI:** 10.1038/sj.bjc.6601055

**Published:** 2003-06-27


British Journal of Cancer (2003) 88(Suppl 1), S11 – S24
© 2003 Cancer Research UK All rights reserved 0007 – 0920/03 $25.00
www.bjcancer.com

### ORAL PRESENTATIONS

### 1.1

THE ROLE OF PARP-1 IN THE CELLULAR RESPONSE TO LOW DOSE RADIATION Anthony Chalmers*, Mick Woodcock, Peter Johnston. Gray Cancer Institute, Northwood UK

**Introduction:** The spectrum of DNA lesions induced by ionising radiation varies according to the dose delivered; hence the relative importance of different DNA repair proteins is also likely to vary with dose. Depletion or inhibition of poly(ADP-ribose) polymerase-1 (PARP-1) is associated with radiosensitisation to doses of 2 Gy and above; that it is activated by single strand breaks and interacts with the base excision repair pathway also indicates a potential role in the response to lower doses. Inhibition of PARP-1 may thus enhance the response of tumours to low dose per fraction and low dose rate radiotherapy protocols. We tested the effect of chemical inhibitors of PARP-1 on low dose clonogenic survival in a range of hamster fibroblast and human tumour cell lines and compared these survival curves with those derived from PARP-1 knockout mouse embryo fibroblasts (MEF).

**Methods:** Clonogenic survival of T98G and U373 human glioma cells, V79 and CHO hamster fibroblasts, and 3T3 MEF PARP-1 knockout and control cells following low-dose irradiation in the presence and absence of chemical PARP inhibitors was measured using a fluorescence-activated cell sorting assay. **Results:** Whilst PARP inhibition induced radiosensitisation of V79, CHO and T98G cells in the 0.05 – 0.5 Gy dose range, there was no such effect in the PARP-1 knockout cells compared with controls.Radiosensitisation to doses greater than 1.5 Gy occurred in all cell lines. **Discussion:** The discrepancy between the effect of chemical inhibition of PARP-1 and deletion of the PARP-1 gene may be explained by upregulation of compensatory low dose repair mechanisms in PARP-1 knockout cells. Another possibility is that inhibition of both PARP-1 and PARP-2 is required for low dose radiosensitisation.

### 1.2

UTILISATION OF MICRO AND MINISATELLITE SEQUENCES FOR INVESTIGATION OF RADIATION INDUCED MUTAGENESIS IN SOMATIC CELLS

Marie Boyd, *Anne Livingstone, Kate Hughes, Lesley Wilson, Anne Marie Clark, Nazia Baig, Susan Ross and Robert J Mairs.

*Department of Radiation Oncology and Targeted Therapy, CRUK Beatson Laboratories, Garscube Estate, Glasgow, G61 1BD.

Characterisation of the mutations induced by radiation is required in order to define human tumours which have a radiation specific origin, to improve radiation protection and to identify patients whose normal tissues are abnormally prone to radiation damage. We have devised a sensitive methodology which allows characterisation of low dose radiation induced mutations in repeat sequences of human somatic cells in culture .We analysed micro- and minisatellite sequences of DNA from clones derived from single irradiated human glioma cells in culture and established a dose response relationship for mutation induction after irradiation with 1-3 Gy.

This methodology was applied to investigate mutagenesis elicited by very low dose radiation (< 1Gy) whose effects are of importance to both cancer radiotherapy and radiation protection, in the UVW cell line which displayed Hyper Radiation Sensitivity (HRS). mini- and microsatellite analysis demonstrated an increased radiation induced mutation rate at radiation exposure doses less than 1Gy. Further, both satellite loci show an 8X increase in radiation induced mutations at 0.3Gy compared to 1Gy. This indicated that enhanced radiation induced mutagenesis accompanied enhanced cell kill at radiation doses below 1Gy. These studies indicate that low dose $\gamma$-irradiation may have implications for radiation protection and have far reaching implications for the scheduling of fractionated radiotherapy, currently employed in cancer treatment.

### 1.3

LOW DOSE HYPER-RADIOSENSITIVITY IN VIVO

Jackie Harney*, Nihal Shah, Susan Short, Michael Joiner, Michelle Saunders. MCRW, Mount Vernon Hospital, UK.

**Introduction** The laboratory phenomenon of "low dose hyper-radiosensitivity" (LDHRS) describes an excess of cell kill at doses < 1Gy relative to that predicted by the LQ model. These biological data have stimulated investigation into the existence of LDHRS *in vivo.* **Methods** Following ethical approval, 2 studies were initiated. Skin was used as a model of normal human tissue. **Study 1:** 24 patients receiving pelvic radiotherapy (RT) were assessed. Once daily doses of ~0.5Gy and >1.0Gy were compared. Biopsies were taken before & during RT & analysed to assess the changes in basal cell density (BCD). **Study 2:** 8 patients with metastatic tumour nodules to skin were evaluated (40 nodules). The nodules were randomised to receive conventionally fractionated RT (1.5Gy/day), or ultrafractionated RT (0.5Gy TDS). Both groups were treated for 12 days (18Gy). Measurements were made on days 0,5,8,12,26 & monthly until regrowth. Biopsies of surrounding skin were also taken to assess changes in BCD & proliferation. **Results: 1:Skin (Study 1):** 19 patients demonstrate a reduction in BCD in the low-dose side compared to the high-dose side when BCD is plotted against dose (significant in 14). Analysis of the whole data set demonstrates a significant reduction in basal cell density in the low dose group. If, however, BCD is plotted against time there is a significant reduction in BCD in the higher dose group. **Study 2:** When BCD is plotted against time, 7/8 patients demonstrate a greater reduction in BCD in favour of the conventional RT arm. A significant reverse fractionation effect was seen in only 1/8 patients. Proliferative response was similar in all patients irrespective of regime. **2: Tumour Nodules:** To date 30 paired nodules have regrown. Analysis of the "radio-resistant" tumours - melanoma & sarcoma -demonstrates significant growth delay in favour of the "ultrafractionated" regime (p = 0.002). **Conclusions:** There is no evidence of LDHRS in normal human skin when regimes of equal dose intensity are compared. LDHRS occurs in "radioresistant" tumours.

### 1.4

PREDICTING THE RESPONSE TO RADIOTHERAPY IN EARLY ONSET BREAST CANCER.

Stewart G Martin*, Rebecca Salmon, Clare Orridge and David AL Morgan. University of Nottingham, Department of Clinical Oncology, City Hospital, Nottingham NG5 1PB

Approximately 1 in 5 female cancers in the UK are breast cancers with ~20% occurring in pre-menopausal women. Young age, <40 years old, is an adverse prognostic factor, notably for local recurrence after breast conserving treatment. Treatment options include surgery and radiotherapy (R/T) to control local disease, and prevent recurrence, and systemic therapies to combat frank or occult metastatic disease. Recent evidence suggests that angiogenesis, as assessed by microvessel density (MVD), may be an independent prognostic factor in breast carcinoma but little is known regarding the prognostic significance of either angio- or lymphangiogenesis in the response of breast cancer patients to post-operative R/T.

A retrospective immunohistological study of biopsies from 82 women with early onset breast cancer (ie age <40 at diagnosis) that underwent breast-conserving surgery and post-operative R/T was undertaken to investigate the role, and prognostic significance, of growth factors (GF's) involved in regulating tumour angio- and lymphangiogenesis (VEGF and VEGF-D respectively). Tumour tissue, and accompanying stroma, were stained using monoclonal antibodies against the GF's and against CD34, to assess MVD. Results, obtained via image analysis, were compared against local recurrence (ie within the treated breast) free survival (RFS), overall survival (OS) and other conventional pathological criteria (tumour stage, grade and vascular invasion).

Of the three markers only VEGF-D was significantly associated with both OS (p=0.01) and RFS (p=0.03). Cox multivariate analysis demonstrates that VEGF-D expression was more significant than conventional criteria indicating the importance of this GF for future studies.

### 1.5

### EVALUATION OF HYPOXIA WITHIN HUMAN PROSTATE CARCINOMA USING QUANTIFIED BOLD MRI AND PIMONIDAZOLE IMMUNOHISTOCHEMICAL MAPPING

Dawn Carnell*, Rowena Smith, Jane Taylor, James Stirling, Peter Hoskin, Anwar Padhani. Marie Curie Research Wing,Mount Vernon Hospital, Northwood, Middlesex, UK.

**Introduction**: The spatial distribution of MRI parameters ($R_2$* & relative blood volume rBV) compared to pimonidazole (an extrinsic marker of hypoxia) stained tissue sections was used to assess the ability of MRI to predict clinically significant prostate cancer hypoxia. This may facilitate localisation of an IMRT boost to potentially radioresistant regions of tumour in the future.

**Methods:** Following local ethical approval, five patients with localised prostate carcinoma were imaged with MRI. Multiple gradient echo (TE 5-75ms) and GRE $T_2$*-weighted images with contrast were used to calculate $R_2$* and rBV maps using a gamma variate fit, pixel by pixel, in a single plane. Areas of fast $R_2$* equivalent to muscle, and rBV greater than fat, were mapped onto a prostate gland outline. 16-24 hours before radical prostatectomy 0.5g/m$^2$ IV pimonidazole was given. Tissue sections in the imaging plane were stained to map tumour (H&E) and hypoxia (pimonidazole). Correspondences between MRI metrics with histology were performed using 5x5mm grid overlays and a 2x2-table analysis for tumour (133 grids) and non-tumour regions (165 grids).

**Results:** Hypoxia was seen within carcinoma and benign prostatic hyperplasia. In tumour 48/60 grids with fast $R_2$* stained positive for pimonidazole and 17/19 with slow $R_2$* stained negative. $R_2$* alone best reflected the oxygenation status of tumours, as defined by pimonidazole, with a sensitivity of 96% and negative predictive value of 89%.

**Conclusion:** $R_2$* best reflected the oxygenation status of tumours supporting the hypothesis that unstimulated BOLD-MRI allows non-invasive mapping of significant hypoxia within human prostate cancer. *This work is supported by Cancer Research UK.*

### 1.6

### GLUT-1 AS A NOVEL TARGET FOR ANTICANCER THERAPY: A COMPARE ANALYSIS USING THE NCI PANEL OF 60 CELL LINES

Andrew Evans,* Stephen Hewitt, Ian J. Stratford, Rachel E. Airley
*School of Pharmacy and Chemistry, Liverpool John Moores University, Byrom Street, Liverpool, UK

Tumours have an increased rate of glucose uptake relative to benign tissue,and tumour hypoxia leads to poor outcome. The increase in glucose uptake observed particularly in hypoxic tumours enables a compensatory switch to anaerobic glycolysis.This effect is mediated chiefly by the HIF-1 regulated glucose transporter Glut-1, which predicts poor prognosis in a wide variety of solid tumours, including cervix, lung and colorectal tumours. Glut-1 is also an intrinsic marker of hypoxia, correlating with $PO_2$ and pimonidazole adduct formation. The prognostic effects of Glut-1, and their relevance to tumour hypoxia, make Glut-1 an interesting target for novel anticancer strategies. For example, it has been found that certain flavonoid tyrosine kinase inhibitors e.g. quercetin, directly bind Glut-1, resulting in a sizeable decrease in glucose uptake that may induce cytotoxicity. In an effort to find possible lead drugs that may mediate their toxicity through Glut-1 binding and inhibition, the level of Glut-1 expression was investigated in the NCI panel of 60 cell lines, using immunohistochemical staining of microarrays containing samples of each tumour cell line. There was a wide variation of Glut-1staining (scored 0-5 according to staining intensity) between cell lines of the panel. Data is currently undergoing analysis, where any correlation between Glut-1 score and cytotoxicity of NCI compounds will be highlighted and investigated further. This work represents a departure from the traditional use of glucose analogues as glycolytic antimetabolites, and instead offers an alternative means of exploiting tumour hypoxia by directly targeting and binding to hypoxia-regulated proteins.

### 1.7

### MNG719, A COMBRETASTATIN PRODRUG TARGETED AT TUMOUR HYPOXIA,

MA Naylor[1], MRL Stratford[1], KB Patel[1], SM Galbraith[1], SW Doughty[2], RM Phillips[3], and SA Everett[1*], [1]Gray Cancer Institute, Mount Vernon Hosp, Northwood, Middlx HA6 2JR. [2]The Pharmacy School, Univ Nottingham, Nottingham, NG7 2RD. [3]Cancer Research Unit, Univ Bradford, Bradford, BD7 1DP.

MNG719, is an indolequinone-based prodrug designed to undergo reductive fragmentation in hypoxia to liberate the anti-vascular agent combretastatin. The impact of MNG719 versus 'free' combretastatin on human umbilical vein endothelial cell (HUVEC) morphology and cytotoxicity were assessed. Target tubulin polymerisation was evaluated by a commercially available assay kit. Substrate specificity and inhibition of recombinant human DT-diaphorase was investigated and results interpreted by parallel molecular modelling. Metabolic studies were performed with HUVEC cell lysates and supersomal cytochrome P450 reductase. Proliferating endothelial cell area is unaffected by MNG719 in air exposed to doses of the prodrug up to 10 μmol dm$^{-3}$ for 30 min whereas under anoxia cell shape reduction or 'shrinkage' is equivalent to doses of combretastatin of less than 1 μmol dm$^{-3}$. Combretastatin completely inhibits tubulin polymerisation at 0.5 μmol dm$^{-3}$ but the prodrug is inactive even at a 40-fold higher concentration. Reductive metabolism of MNG719 by HUVEC cells and cytochrome P450 reductase under anoxia liberates combretastatin which is inhibited by air and is reflected in the comparative anoxia versus air cytotoxicities. Despite the 'bulkiness' of MNG719 the prodrug can dock in the active site of DT-diaphorase where reductive fragmentation results in enzyme inhibition. The results are consistent with MNG719 'redox-cycling' in air but undergoing reductive fragmentation under anoxia, mainly by cytochrome P450 reductase, to release combretastatin. Prodrug-activation is 'oxygen-sensitive' and future work will seek to modify the rate of reductive fragmentation to control hypoxia-selective drug delivery. *This work is supported by Cancer Research UK*

### 2.1

### IDENTIFICATION OF TUMOUR SUPPRESSOR GENES IN CHILDHOOD MEDULLOBLASTOMA BY PROMOTER HYPERMETHYLATION PROFILING

Janet C. Lindsey, Meryl E. Lusher, Andrew D.J. Pearson, David W. Ellison and Steven C. Clifford.

Northern Institute for Cancer Research, University of Newcastle, Newcastle upon Tyne, UK.

Medulloblastoma (MB) is the most common malignant brain tumour of childhood, however the molecular pathogenesis of the majority of MBs is not understood. The epigenetic silencing of tumour suppressor gene (TSG) expression by promoter hypermethylation has emerged recently as an important mechanism of TSG inactivation. To assess the significance of aberrant promoter methylation events in MB, and to identify critical genes in its pathogenesis, we profiled the methylation status of eleven candidate TSGs (*p14$^{ARF}$, p15$^{INK4b}$, p16$^{INK4a}$, Caspase 8, HIC-1, EDNRB, TIMP-3, TP73, TSLC1, RIZ-1 and RASSF1A*) in MBs (11 cell lines, 44 primary tumours) and the normal cerebellum. Our data show that extensive hypermethylation of the *RASSF1A* promoter is a tumour-specific event, which occurs in the majority (~90%) of MBs. In MB cell lines, *RASSF1A* methylation was associated with epigenetic transcriptional silencing. Moreover, total *RASSF1A* methylation was observed in the absence of gene deletion or mutation, indicating that *RASSF1A* inactivation in MB occurs by bi-allelic epigenetic mechanisms. Increased tumour-specific methylation was also observed for *HIC-1* (39%) and *Caspase 8* (32%), in both cases occurring against a background of normal tissue-specific methylation in the cerebellum. These data demonstrate that aberrant promoter methylation events are a significant feature of MB development, and identify putative MB TSGs. Epigenetic *RASSF1A* inactivation represents the most common defect detected to date in MB, emphasising the importance of its further investigation in this disease.

© 2003 Cancer Research UK

**2.2**

ANTISENSE OLIGONUCLEOTIDE TARGETING OF RAF-1: IMPORTANCE OF mRNA EXPRESSION LEVELS IN DETERMINING GROWTH RESPONSE

Simon P Langdon*[1], Peter Mullen[1], Fiona McPhillips[1], Kenneth MacLeod[1], Brett Monia[2] and John F Smyth[1]. [1]Cancer Research UK Edinburgh Oncology Unit, Western General Hospital, Edinburgh EH4 2XR ; [2]ISIS Pharmaceuticals, Carlsbad, USA.

Raf-1 is a member of the Raf serine/threonine kinase family which consists of Raf-1 (c-Raf), A-Raf and B-Raf. It is a key intermediate in the Ras/Raf/MEK/ERK pathway which relays extracellar signals to the nucleus. Over 90% of ovarian carcinomas express Raf-1 protein, with high levels correlating with poor survival (McPhillips et al, Br J Cancer, 85, 1753,2001). In this study we have used a series of 15 ovarian cancer cell lines to help identify determinants of sensitivity to anti-Raf-1 antisense oliogonucleotides (ASOs). Growth inhibition (at 72h) varied from 100% down to 12% across this series of cell lines after treatment (200 nM for 3h) with either fully phosphorothioated ASO, ISIS 5132, (TCCCGCCTGTGACATGCATT) or a further (2'methoxyethyl) modified ASO, ISIS 13650. The degree of growth inhibition was not associated with level of Raf-1 protein expression, intracellular uptake of ASO or extent of Raf-1 protein inhibition after ASO treatment (61-99% inhibition). However, growth inhibition was significantly (p=0.019, Mann-Whitney) higher in cell lines with a higher proportion of Raf-1 mRNA (relative to total Raf mRNA i.e. Raf-1 + A-Raf + B-Raf) suggesting that greater utilisation of Raf-1 rather than other Raf isoforms was important. Consistent with this, growth inhibition was also significantly (p=0.04, Mann-Whitney) higher in cell lines which demonstrated greater (>2-fold) Raf-1 kinase activation after growth factor (1nM TGF α) stimulation. These results indicate that ovarian cancer cell lines demonstrate variable growth sensitivity to anti-Raf ASOs but sensitive tumors can feasibly be identified.

**2.3**

THE BIOLOGICAL ACTIVITY OF ENDOTHELIAL HEPARAN SULFATE IN SEROUS ADENOCARCINOMA OF THE OVARY

Melissa K. Whitworth*, Godfrey Wilson, Richard Slade, John Gallagher, Alan Rapraeger and Gordon Jayson. Cancer Research UK Department of Medical Oncology, Christie Hospital NHS Trust, Wilmslow Road, Manchester, M20 4BX, UK.

Fibroblast growth factor-2 (FGF2) is a potent angiogenic factor, which requires heparan sulfate proteoglycans (HSPGs) for its biological activity. HSPGs mediate stable high affinity binding of FGF2 to its receptor tyrosine kinases (FR). The aim of this study was to investigate the biological activity of epithelial ovarian cancer (EOC) endothelial HS with respect to the activation of FGF2.

We examined the ability of HSPGs to promote FGF2/FR binding using a soluble FR fusion construct (FR1-AP) (1). This encodes the extracellular domain of FR1 linked to alkaline phosphatase (AP). In addition we developed IHC techniques to assess the expression of endogenous FGF2, FR1 and FR2 and a doublestain technique to assess HS chain expression on ECs.

FGF2 binding was HS chain dependent. HSPGs capable of supporting FGF2 binding are present on ECs and throughout the stroma and epithelium. FGF2/FR1-AP fusion complex localisation is significantly different from FGF2 localisation (p<0.0005), suggesting that HS chains held in an active configuration are located primarily on ECs. FR1 and FR2 have a variable distribution within EOC specimens and are found mainly on stromal and epithelial cells. The double stain technique confirms that HS chains are present on ECs.

We have shown for the first time that the HS expressed by ECs in EOC has the capacity to activate FGF2, an angiogenic protein. This identifies HS as a potential target for anti-angiogenic therapies. (1) Chang et al. (2000) Faseb J. 14, 137

**2.4**

DYSREGULATION OF CELL CYCLE CONTROL PROTEINS DURING CERVICAL GLANDULAR CARCINOGENESIS

Alaa A. El-Ghobashy* [1], Abeer M. Shaaban [1], Jonathan Herod [2], Jenny Innes [1], Wendy Prime, Simon Herrington [1]
[1]Department of Cellular and Molecular Pathology, the University of Liverpool and [2]Liverpool Women's Hospital, Liverpool, UK

A putative pathway for cervical glandular carcinogenesis exists from normal glands through cervical glandular intraepithelial neoplasia (CGIN) to invasive adenocarcinoma. Cyclins and CDK-inhibitors are known to play a crucial role in regulating cell cycle. We aimed to identify biological markers that define early cervical glandular neoplasia and to differentiate CGIN from their benign mimics. Paraffin-embedded sections of normal cervix (Group: 1, n=11) edometriosis/TEM (Group: 2, n=19), CGIN (Group: 3, n=33) and invasive adenocarcinoma (Group: 4, n= 28) were stained with p16, cyclins A, B, D and E. A high level of p16 expression was found in CGIN (mean: 81.4%) and invasive adenocarcinoma (84.4%) when compared with normal endocervix (2.3%). Levels of cyclin A, cyclin B and p16 in CGIN were significantly higher than those in group 2 (*P*= 0.05, <0.001 and <0.001 respectively). A progressive increase in cyclin A and B expression occurred from normal cervix to invasive adenocarcinoma. The latter expressed higher levels of cyclin A and B when compared with CGIN (*P*<0.001). Cyclin E expression increased from group 1 to 2 (*P*=0.03) with no significant further increase. Changes in Cyclin D expression were unremarkable. Our data highlight that p16 could be used as a marker for cervical glandular neoplasia. Concurrent expression of cyclin A, cyclin B and p16 can help distinguish between CGIN and TEM/endometriosis.

**2.5**

ASSOCIATION OF VITAMIN D RECEPTOR GENE POLYMORPHISMS WITH BREAST CANCER RISK AND PROGRESSION.

Michelle Guy*, Lorraine C. Lowe, Janine L. Mansi, Rosalind Given-Wilson, Valerie Thomas and Kay W. Colston.
St George's Hospital Medical School, London, SW17 0RE, UK.

**Aims**: The steroid hormone vitamin D is thought to protect against breast cancer (BC). Vitamin D exerts its cellular effects by binding to the vitamin D receptor (VDR). The VDR has several polymorphic sites, resulting in differing genotypes that may alter susceptibility to BC. We have investigated whether specific polymorphisms in the VDR gene are associated with BC risk in a UK Caucasian population.

**Methods**: Female BC patients (n=411), and control women with a negative screening mammogram (n=427) were recruited, and their VDR genotypes were determined.

**Results**: The 3′ VDR polymorphism *Bsm*I and a variable length Poly A sequence were both significantly associated with BC risk; odds ratio *bb* vs *BB* genotype = 1.74, (*P*=0.0096); odds ratio LL vs SS = 1.70, (*P*=0.0146). A 5′ VDR gene variant, *Fok*I was not associated with BC risk when analysed in isolation (*P*=0.31). However, *Fok*I modulated the increased risk associated with the *bb/*LL genotype such that possession of one or more *F* alleles together with the *bb/*LL genotype augmented the risk of BC. Furthermore, the highest proportion of *bb* (53%) and *FF*LL/*Ff*LL (52%) genotypes occurred in women with distant metastatic BC, and 73% of established cell lines derived from BC metastases (n=15) displayed the *bb/*LL genotype.

**Conclusions**: VDR polymorphisms are associated with BC risk and progression. This research has implications for the monitoring and treatment of women at risk of BC, and for those likely to develop metastasic disease. Future research will investigate the effect of genotype on VDR function.

© 2003 Cancer Research UK

### 2.6

ESTROGEN CAN MODULATE ESTROGEN RECEPTOR-INDEPENDENT SURVIVAL PATHWAY IN HUMAN BREAST CANCER. Kheng T. Lim*, Arnold D.K. Hill, Enda W. McDermott, Niall J. O'Higgins, Leonie S. Young. Department of Surgery, St. Vincent's University Hospital and the Conway Institute for Biomolecular and Biomedical Research, University College Dublin, Dublin 4, Ireland.

In breast cancer, steroids in particular estrogen, functioning through its receptors (ER) contribute to tumour progression by regulating the transcription of target genes. Recent studies however suggest that estrogen may also up-regulate the mitogen-activated protein kinase (MAPK) pathway, mimicking the effect of growth factors such as epidermal growth factor (EGF). This clinical observation suggests that estrogen may be able to mediate some of cell survival properties through an ER-independent mechanism.

The ER-positive MCF-7 and the ER-negative SKBR3 human breast cancer cell lines were incubated in the presence and absence of EGF and estrogen with or without G-protein coupled receptor (GPCR) inhibitor, pertussis toxin and EGF receptor inhibitor, AG 1478. Phospho-c-raf, phospho-Erk, phospho-cdc2 and survivin expression were detected using western blotting techniques.

In MCF-7 and SKBR3 human breast cancer cells, we have found that EGF and estrogen stimulation rapidly increased the phosphorylation of c-raf, Erk and subsequently an increase in cdc2 phosphorylation and an up-regulation of survivin expression. A further increase was noted in the presence of EGF in combination of estrogen. Moreover, estrogen induced a translocation of phospho-cdc-2 from the cytosol to the nucleus indicating activation of the phospho-cdc-2/survivin complex. The effects of EGF on phosphorylation of c-raf, Erk, cdc2 and survivin were attenuated by AG1478. The effects of estrogen on phosphorylation of c-raf, Erk, cdc2 and survivin were attenuated by pertussis toxin and to the lesser extent by AG1478.

These observations implicate estrogen in the MAPK cell survival mechanism and survivin anti-apoptotic pathway. Elucidation of ER-independent estrogen survival pathways may in part explain clinical observations of steroid therapy resistance in breast cancer.

### 3.1

SRC-1 EXPRESSION IS ASSOCIATED WITH ENDOCRINE RESISTANCE IN BREAST CANCER

Fergal Fleming*, Arnold Hill, Enda Mc Dermott, Niall O'Higgins, Leonie Young

Department of Surgery, St Vincent's University Hospital and Conway Institute of Biomolecular and Biomedical Research, University College Dublin, Ireland

ER-α and ER-β function as transcription factors to modulate genes relevant to breast cancer progression. Both interact with nuclear regulatory proteins to enhance or inhibit transcription. Following ethical approval ER-α, ER-β, the co-activator SRC-1, and the co-repressor SMRT were localized within breast tissue by immunohistochemistry, and their spatial co-expression assessed by immunofluoresence. The expression of ER-α, ER-β and the coregulators was correlated with clinicopathological features. The ability of β-estradiol and 4-hydroxytamoxifen (4-OHT) to modulate protein expression of ER-α, ER-β, SRC-1, and SMRT in primary breast cancer cell cultures was assessed by immuno-blotting. SRC-1 was found to be significantly associated with nodal positivity and resistance to endocrine treatment. There was up-regulation in ER-β and the co-regulator protein expression in the presence of β-estradiol. 4-OHT induced an increase in ER-α and SMRT, whereas it decreased or unaltered the protein expression of ER-β and SRC-1. Associations between SRC-1 expression and recurrence suggest that co-activators may impact on the clinical phenotype of the tumour. The differential regulation of the ER-isoforms and the co-regulators by β-estradiol and 4-OHT provides evidence that co-regulatory proteins may have a role in determining the response of breast cancer cells to estrogen and tamoxifen.

### 2.7

HER2 OVEREXPRESSION CONFERS RELATIVE RESISTANCE TO ZOLEDRONIC ACID *IN VITRO*

Lisa M Pickering*, Jon P Coxon, Janine L Mansi, Kay W Colston
St. George's Hospital Medical School, London. UK. SW17 0RE.

Her2 receptor overexpression confers an increased risk of breast cancer recurrence and is associated with relative resistance to many endocrine and chemotherapeutic agents. Bisphosphonates, particularly potent third generation compounds such as zoledronic acid (ZA), limit skeletal morbidity in patients with advanced breast cancer. We have previously shown that ZA induces apoptosis in her2 non-overexpressing breast cancer cell lines and limits their capacity to adhere to mineralised matrices. This study aimed to investigate whether her2 receptor overexpression attenuates sensitivity to these ZA-induced anti-cancer effects.

Adhesion was assessed by seeding viable cells onto extracellular matrices for 24 hours. Adherent cells were then washed, fixed, stained and counted. Induction of apoptosis was assessed by the cell death ELISA and MTS cell viability assays.

Forced her2 receptor overexpression in the non-malignant breast epithelial cell line Hb4a resulted in a 2-fold increase in their capacity to adhere to mineralised matrix. Treatment with 1nM-50μM ZA for 24h resulted in a significant reduction in adhesion of two non-her2 receptor overexpressing cell lines cells, with a reduction in adhesion of 50-85% in MCF-7 cells and 35-80% in MDA-MB-231 cells. However the her2 receptor overexpressing SKBr3 cell line experienced only 16% reduction in adhesion with ZA treatment. Furthermore, whilst ZA induced apoptosis in all three cell lines, a significantly lower percentage of SKBr3 cells than either MCF-7 or MDA-MB-231 cells underwent apoptosis.

In conclusion, these results support the observation that her2 receptor overexpression is associated with increased metastatic potential and suggest that it confers relative resistance to ZA.

### 3.2

ABERRANT EXPRESSION OF INTERLEUKIN-7 (IL-7) AND ITS RECEPTOR (IL-7R) IN BREAST CANCER AND THE ASSOCIATION WITH PROGNOSIS. Mahir A A Al-Rawi*, Robert E Mansel and Wen G Jiang. Department of Surgery, University of Wales College of Medicine, Cardiff CF14 4XN UK

IL-7 is known to induce the differentiation and proliferation of some leukaemias and lymphomas. Although it has been implicated in the process of lymphangiogenesis, Little is known about its role in solid tumours including breast cancer. We studied the expression of IL-7 and its receptor, IL-7R, in a cohort of patients with breast cancer.

Tumour (n=122) and normal (background) tissues (n=41) from patients with breast cancer (median follow up 72.2 months) were analysed for the level of mRNA of IL-7 and IL-7R, using quantitative RT-PCR, and protein using Western blotting and immunohistochemistry. The results were analysed according to tumour grade, nodal involvement, patients survival rate and prognosis (using the Nottingham Prognostic Index).

The expression of IL-7 was higher in the cancer tissues compared to the background tissues. IL-7 was significantly higher in grade 3 tumours compared to grades 2 and 1 ($P$=0.03). Ductal carcinomas highly expressed IL-7 compared with lobular tumours ($P$=0.08). Furthermore, IL-7 expression was significantly higher in the node positive group compared to the node negative patients (P=0.03). Number of copies of IL-7 was higher in the worst prognosis group (NPI3) compared to the good prognosis group (NPI1) (P=0.004). Additionally, patients who died from breast cancer had significantly higher IL-7 expression in their tumours compared with the survivals ($P$=0.03). The expression of IL-7R was also significantly elevated in node positive tumours and in patients with NPI3.

This is the first study to show that IL-7 expression is associated with tumour grade, nodal involvement and prognosis in breast cancer. Aberrant expression of IL-7 and IL7R in breast cancer may be involved in the progression and spread of breast cancer.

© 2003 Cancer Research UK

3.3

MECHANISMS OF TELOMERASE INHIBITION BY NOVEL POLYCYCLIC ACRIDINES

Jennifer C. Cookson*, Robert Heald, Laurence Hurley, Malcolm F. G. Stevens. *Cancer Research Laboratories, School of Pharmaceutical Sciences, University of Nottingham, UK

A series of novel polycyclic acridines inhibit telomerase in the TRAP assay ($^{tel}IC_{50}$ ~ 0.3μM) and are highly promising potential new anti-cancer agents. The lead compound, RHPS4 (4,11-difluoro-6,8,13-trimethyl-8H-quino[4,3,2-kl]acridinium methosulfate) exhibits anti-proliferative effects, induction of senescence and reduction in telomere length; cell extracts incubated with RHPS4 show reduced telomerase activity. Modelling, biophysical and NMR data suggest the compounds stabilise G-quadruplex structures such as those formed in the telomeres and possibly the *c-myc* oncogene promoter.

The *c-myc* gene is capable of activating telomerase by increasing expression of the catalytic subunit of telomerase (TERT). We investigated whether the polycyclic acridines might stabilise a quadruplex structure in the *c-myc* promoter and reduce cellular expression of *c-myc* and possibly in turn *hTERT*. A cell-free, polymerase stop assay revealed potent stabilisation by the compounds of a G-quadruplex structure in the *c-myc* promoter sequence. Effects on *c-myc* expression in vitro were evaluated by RT-PCR with members of the series eliciting a modest reduction in *c-myc* and *hTERT* mRNAexpression in cancer cell lines. However, the reduction in *hTERT* expression does not appear to be mediated exclusively through a reduction in *c-myc* and the pattern of *c-myc* down-regulation in cells does not correlate with G-quadruplex stabilisation in the stop assay. Thus we conclude that the anti-proliferative and telomerase inhibitory effects of these compounds are not significantly mediated through a reduction in *c-myc* expression, but more likely through direct interaction with the telomeres.

3.4

IDENTIFICATION OF pVHL-INTERACTING PROTEINS AND THEIR ROLE IN PHEOCHROMOCYTOMA DEVELOPMENT

James C. Cooke* and Steven C. Clifford.
Northern Institute for Cancer Research, The Medical School, University of Newcastle-upon-Tyne, NE2 4HH, U.K.

von Hippel-Lindau disease (VHL) is a dominantly inherited familial cancer syndrome caused by mutations of the *VHL* tumour suppressor gene (TSG). VHL predisposes to the development of tumours including renal cell carcinoma (RCC), hemangioblastoma (HAB) and pheochromocytoma (PHE). Distinct patterns of tumour development and genotype-phenotype correlations exist in VHL disease, suggesting multiple and tissue-specific functions for pVHL, the *VHL* TSG protein product. pVHL plays a critical role in the regulation of hypoxia inducible factor-1α (HIF-1α) and cellular oxygen sensing. We recently showed that disruption of HIF-1α regulation by disease-associated pVHL mutants correlated with RCC and HAB predisposition in VHL disease, however HIF-1α de-regulation was not associated with PHE susceptibility.

To investigate further pVHL functions that may play a role in VHL-associated PHE development, we have performed a yeast-2-hybrid screen against a PHE-representative cDNA library to identify putative pVHL-interacting proteins. This assay initially identified 100 potential pVHL-interacting clones. Twenty of these were characterised in detail, and 10 unique binding species were identified which represented in-frame protein coding sequences. These included 6 uncharacterised cDNAs and 4 known proteins including the zinc finger protein, Zbrk-1, and eNOS-interacting protein. The specificities of all ten interactions were confirmed in yeast using small-scale yeast-2-hybrid assays.

The ten pVHL-interacting proteins identified are now the focus of further investigations to establish their *in vitro* and *in vivo* significance, and any role they may play in PHE development.

3.5

THE ANTI-APOPTOTIC MCL-1 PROTEIN PLAYS A KEY ROLE IN B-LYMPHOMA CELL SURVIVAL

Jorg Michels *, Eric Marcusson, Peter W.M. Johnson, Graham Packham, Cancer Research UK, Cancer Sciences Division, University of Southampton, Southampton, UK.

Introduction: Mcl-1 is an anti-apoptotic Bcl-2 family protein that is frequently expressed in malignant lymphocytes. Mcl-1 is often down regulated early during apoptosis. Here we have used anti-sense techniques to determine the importance of Mcl-1 expression in controlling B-cell lymphoma survival.

Methods: Akata6 cells (B-cell lymphoma cell line) were transfected with Mcl-1 antisense or missense oligonucleotides (ODN) using electroporation in the presence or absence of ZVAD-fmk. Expression of Bcl-2 family proteins (Bcl-2, Bcl-$X_L$, Mcl-1), caspases and PARP was determined by immunoblotting.

Results: Transfection of Mcl-1 antisense, not missense ODN resulted in early down regulation of Mcl-1 protein in contrast to related molecules, Bcl-2 and Bcl-$X_L$. Down regulation of Mcl-1was followed by activation of caspases and cleavage of PARP, a caspase substrate. Activation of caspases by the Mcl-1 antisense ODN was accompanied by the appearance of a 28kDa Mcl-1cleavage fragment, suggesting that Mcl-1 is also cleaved by caspases during apoptosis. The 28 kDaMcl-1 cleavage product was also detected during cisplatin-induced apoptosis of Akata6 cells. Treatment with ZVAD-fmk prevented caspase activation and Mcl-1 cleavage induced by Mcl-1 antisense ODN, but not the initial decrease in Mcl-1 protein levels preceding these events.

Conclusions: These data demonstrate that Mcl-1 is a key survival molecule for B lymphoma cells. Down regulation of Mcl-1 by antisense ODN is sufficient to activate apoptotic pathways and caspase mediated cleavage of Mcl-1 may function as a positive feedback loop to ensure efficient cell killing.

3.6

MOLECULAR GENETICS, PATHOLOGY & PHYSIOLOGY OF GLIOMAS TREATED BY PCV CHEMOTHERAPY

Carol Walker[+*], Daniel DuPlessis[#], Kathyrn A. Joyce[+], Trevor Smith[#], Sobhan Vinjamuri[†], Brian Haylock[∞], David Husband[∞], Aditya Shenoy[∞], John Broome[#] and Peter C. Warnke[#] [+]Clatterbridge Cancer Research Trust and [∞]Clatterbridge Centre for Oncology, Clatterbridge Hospital, Bebington, Wirral, CH63 4JY; [#]Walton Centre for Neurology and Neurosurgery and [†]Royal Liverpool University Hospital, Liverpool, UK.

The diagnosis and treatment of oligodendroglial neoplasms is currently highly controversial. Loss of chromosomes 1p and 19q may be predictive of chemosensitivity, while tumour physiology influences drug delivery and therapeutic responsiveness.

In a prospective study of 65 gliomas treated with PCV chemotherapy at a single treatment centre, laser capture microdissection and microsatellite analysis was used to determine allelic losses. 11/18 OII, 5/18 OAII, 9/10 OIII, 6/18 OAIII and 0/1 GBMO had loss of both 1p36 and 19q13. 3 OII, 7 OAII, 3 OIII, 9 OAIII and 1 GBMO had loss of 17p13 and 5 OIII and 7 OAIII had losses in chromosome 10. Inter or intratumoral heterogeneity of histological subtype seen in 9 cases, was not reflected in genetic heterogeneity. Comparison of pre-treatment molecular genetics and physiology data suggests that tumours with 1p36/19q13 loss are more likely to have increased uptake of $^{201}$Tl. For a subset of cases, pre and post therapy *in vivo* imaging data, including $^{1}$H MR Spectroscopy, rCBV, $^{18}$FDG-PET and $^{201}$ Tl-SPECT is being used to assess radiological responses. For example, a hypermetabolic anaplastic tumour with loss of 1p36/19q13 responded completely to PCV, while a hypermetabolic anaplastic tumour with loss of 17p13 and mutant p53 progressed. Continued clinical follow–up will elucidate the complex physiological and molecular factors that may influence chemosensitivity in oligodendroglial neoplasms.

© 2003 Cancer Research UK

3.7

MONITORING OF IMMUNOLOGICAL CHANGES AFTER ADMINISTRATION OF A WHOLE CELL ALLOGENEIC PROSTATE CANCER VACCINE.

Whelan M.A.*[1], Michael A.[2], Quatan N.[2], Russell N.[1], Whelan J.[1], Wushishi F.[1] & Pandha H.[2]
[1]Onyvax Ltd. & [2]Dept. of Oncology, St. George's Hospital Medical School, Cranmer Terrace, London, SW17 0RE, UK.

Allogeneic whole cell vaccines are a practical approach to immunotherapy. They do, however, present unique challenges for clinical trial monitoring since their multivalent antigenic nature makes it impractical to examine specific immune responses.

We have examined cytokine secretion by peripheral blood lymphocytes (pbl) gathered from a Phase I/II study in hormone refractory prostate cancer. Patients were shown to be free of bone metastases and immunocompetent on entry. Each individual received a mixture of three allogeneic prostate lines plus BCG adjuvant on weeks 0 and 2. Patients received cells alone on week 4 and monthly thereafter for one year. Blood samples were taken at visit and weekly on weeks 12-16 and 32-36.

Pbl were thawed and stimulated overnight with either PHA/ionomycin or LPS. Cytokine levels were measured in the supernatants by cytometric bead array (CBA) and the producing cells' RNA used for real-time PCR with absolute quantitation.

Data from 15 subjects initially showed significant elevations in $T_H1$ cytokines in patients with marked increases in PSA doubling times. As the trial progressed, this phenotype became mixed $T_H1$ and $T_H2$. Patients with no PSA response showed either a complete lack of cytokine detection or elevated $T_H2$ cytokines. Interestingly, protein and cDNA levels correlated well up to week 16 and then cDNA levels appeared depressed compared to protein.

3.8

ANTI-CANCER DRUG INTERACTION WITH CYTOCHROME P450 CYP1B1

Morag C E McFadyen*, William T Melvin, Graeme I Murray
Department of Pathology, University of Aberdeen, Aberdeen, AB25 2ZD UK.

The major goal of cancer research is the development of therapeutic agents specifically aimed at tumour cells. One mechanism potentially amenable to chemotherapeutic intervention involves cytochrome P450 CYP1B1. Our research has established the concept of over-expression of individual forms of P450 in particular CYP1B1 ina range of solid tumours. Moreover our *invitro* studies have demonstrated the presence of metabolically active CYP1B1 and cytochrome P450 reductase in tumour samples. We have previously identified several anti-cancer drugs (docetaxel, paclitaxel, mitoxantrone and flutamide) as substrates for CYP1B1. Furthermore, our *in vitro* studies have shown that the presence of CYP1B1 reduces the efficacy of docetaxel. In this study we used expressed human CYP1B1 in a competitive microassay involving the deethylation of ethoxyresorufin to resorufin to extended our screen of anti-cancer drugs and identify which ones interact with CYP1B1, by the change in resorufin production over time. Our findings to date indicate that a range of structurally diverse anti-cancer drugs interact with CYP1B1 (melphalan, bleomycin, methotrexate, altretamine, ellipticine, resveratrol, carmustine, dactinomycin, raltitrexed, epirubicin, and mitomycin C). Several of these drugs have been further characterised to determine their mechanism of interaction with CYP1B1. The over-expression of CYP1B1 in tumour cells and interaction of this P450 with anti-cancer drugs highlight CYP1B1 as an important P450 in tumour cells.

Acknowledgement: This research was funded by Cancer Research UK and the Gray Fund.

4.1

THE TREATMENT OF WILMS' TUMOUR: RESULTS OF THE UNITED KINGDOM CHILDREN'S CANCER STUDY GROUP THIRD WILMS' TUMOUR STUDY.

Christopher Mitchell [(1)] Rosemary Shannon, Gordan, M Vujanic, Anna Kelsey, Peter Gornall Jenny Walker, Richard Grundy, Catherine Owens, Roger Taylor Kathy Pritchard Jones, John Imeson, Helen Middleton, Carolyn Hutton, Jon Pritchard, For the United Kingdom Children's Cancer Study Group.
1. Paediatric Oncology, Oxford Radcliffe Hospital, Oxford OX 9DU.

**Background:** The prognosis for children with Wilms tumour has improved steadily over the past four decades so that it is now possible to focus on treatment refinement with a view to reduction of short and long-term side effects. One remaining area of contention is the timing of surgical resection. It has been suggested that the use of preoperative chemotherapy might reduce the risks of surgical intervention and might, by identifying tumours that are chemosensitive, reduce the total burden of therapy needed by patients fortunate enough to have a responsive tumour.

**Methods:** Children with nonmetastatic renal tumours that were judged to be surgically resectable were randomly assigned either to immediate nephrectomy or to percutaneous biopsy followed by six weeks of chemotherapy with actinomycin D and vincristine followed by delayed nephrectomy. In both arms postoperative therapy was dictated by the tumour stage assigned at the time of nephrectomy.

**Results:** During the period October 1991 to March 2001) of the 607 eligible patients, 205 were randomised. Of this group 21 were subsequently found to have non-Wilms histology. There were 94 patients who had immediate nephrectomy and 90 patients who had preoperative chemotherapy. Analysis of the stage distribution in the two arms shows a highly significant improvement in the stage distribution for the preoperatively treated group (stage I: 66.3 vs 55.3, stage II: 23.3 vs 14.9, stage III: 10 vs 29.8%). EFS and OS were equivalent between the two arms. There was also a reduction in peroperative tumour rupture and other surgical complications.

**Conclusions:** Six weeks of preoperative therapy with vincristine and actinomycin D for patients with Wilms tumour identifies patients with chemoresponsive tumours and leads to a more favourable stage distribution, permitting a reduction in the overall burden of their therapy without compromising the chances of cure.

4.2

LOW FREQUENCY OF CONSTITUTIONAL *WT1* MUTATIONS IN CHILDREN WITH SPORADIC WILMS TUMOUR.

Suzanne Little*, Sandra Hanks, Linda King-Underwood, Liz Rapley, Naz Rahman, Kathy Pritchard-Jones on behalf of the UK Children's Cancer Study Group (UKCCSG).

**Aim:** To ascertain the frequency of constitutional *WT1* mutations in unselected UK patients with sporadic Wilms tumour (WT) and investigate heritability.

**Methods:** Constitutional DNA from 294 unselected patients with sporadic WT treated at 7 UKCCSG centres was screened for mutations in *WT1* by heteroduplex analysis. 11 samples in which >20% of PCR fragments failed were excluded. Suspected mutations were confirmed by sequencing and analysed in parental samples to investigate inheritance. The cohort included 264 unilateral WT, 18 bilateral and 1 extrarenal.

**Results:** Six children had constitutional *WT1* mutations: a nonsense and a splice site mutation in exon1, a frameshift in exon3 and three nonsense mutations in the zinc finger region (R301X and two identical R390X mutations), the latter are recognised somatic and constitutional mutations in Wilms tumour. These mutations were not present in any of the 9 parents tested. The *WT1* mutation group had a young median age at diagnosis (13.8m), 4 were female, 2 were male. Only 1/6 had bilateral tumours, all were favourable histology. Only 1/6 had congenital abnormalities (ptosis, bilateral cryptorchidism). None had family history of renal disease or early onset cancers.

**Conclusions:** This study demonstrates that a small proportion (2.12%: 95%CI 0.44%-3.80%) of children with apparently sporadic Wilms tumour carry constitutional *WT1* mutations. Only 2/6 had features suggestive of genetic predisposition. These findings have implications for the counselling of patients with Wilms tumour and their parents.

We thank all UKCCSG centres who contributed to this study and the families for their participation.

© 2003 Cancer Research UK

### 4.3

MAGNETIC RESONANCE SPECTROSCOPY (MRS) TO STUDY CHEMOTHERAPY RESPONSE IN A PAEDIATRIC EMBRYONAL RHABDOMYOSARCOMA (Rd) MODEL.

Sucheta J Vaidya*, Yuen-Li Chung, Geoffrey S Payne, Martin O Leach, John R Griffiths, C Ross Pinkerton.
Royal Marsden NHS Trust, Institute of Cancer Research, Surrey and St George's Hospital Medical School, Tooting, UK.

**Aims**: *In vivo* $^{31}$P MRS can be used to obtain information non-invasively on tumour bioenergetics (NTP/inorganic phosphate (Pi) ratio), intracellular pH, and membrane metabolism (measuring phosphorylcholine (PC) and phosphorylethanolamine (PE)). The aim of this study was to investigate these as surrogate markers for tumour response after ifosfamide (Ifos) therapy in Rd xenografts with a view to translating these pharmacodynamic markers to clinical studies.

**Methods**: Rd xenografts were grown in nude mice. When tumour size reached 582 ± 47 mg, *in vivo* localised $^{31}$P MRS was performed before and 7 days after treatment with saline (control, n=5) or Ifos (250mg/kg, n =11). Tumours were then extracted and analysed using complementary high resolution $^{31}$P MRS *in vitro*.

**Results:** At 7 days, tumour volume changed by 7±7% after Ifos compared with an increase of 102± 19% after saline (p = 0.006). (PE+PC)/Pi (p = 0.03) and β-ATP/Pi (p=0.04) ratios were significantly increased in Ifos-treated tumours, whereas there were no significant changes in controls. PE was also increased in the extracts of Ifos-treated tumours compared with control (p = 0.005) The *in vitro* finding confirmed the in vivo data and showed that the rise of the (PC+PE)/Pi ratio *in vivo* was due to increased PE, with PC and Pi unchanged.

**Conclusions**: Significant changes in tumour bioenergetics and membrane metabolism were found after chemotherapy. This suggests measurements with $^{31}$P MRS could be used as a useful non-invasive surrogate marker in clinical studies.

### 4.4

THE INCIDENCE OF CANCER IN ADOLESCENTS AND YOUNG ADULTS IN SOUTH EAST ENGLAND. AN ANALYSIS OF THAMES CANCER REGISTRY DATA 1998-2000

Murphy DM*, Dolbear C, Møller H, Whelan JS, Pinkerton CR
*Department of Paediatrics, The Royal Marsden Hospital, Sutton, SM2 5PT

Cancer patients aged 15-24 years have distinct needs. They are recognised as a special group in The Cancer Plan. High quality cancer statistics are vital for service planning.

Anonymised individual level data for the years 1998-2000 were obtained from Thames Cancer Registry. For registered cancers in the age range 10-24 years frequency tables were produced. Tumours were categorised using the new Birch-Alston (Manchester) classification. A total of 1,394 tumours were identified over approximately 7.7million person years at risk. Annual incidence rates per million were calculated based on mid-year population estimates for the study region. Age specific incidence rates for each 5-year age group: 10-14, 15-19 and 19-24 years were calculated. Direct standardisation to the world population was used to calculate an age-standardised rate. A crude analysis of service requirement was formulated using registry-derived figures for uptake of chemotherapy, radiotherapy and surgery.

Overall rates in the three age groups were 97.3, 167.8 and 268.2 per million person years respectively. Lymphoma was the commonest diagnosis in all age groups. Our results closely mirror nationally published data. We have described a population of teenagers and young adults, using a novel classification scheme that helps target studies in young cancer patients.

### 4.5

CELL DEATH FOLLOWING EXPOSURE OF ESFT CELLS TO bFGF IS EFFECTED BY A p38-DEPENDENT UP-REGULATION OF DEATH RECEPTORS.

James Boyne* and Susan Burchill.
Cancer Research UK, St James's University Hospital, Leeds.

The aim of this study was to further characterise the role of death receptors in bFGF-induced cell death in Ewing's sarcoma family tumour (ESFT) cell lines, and examine the hypothesis that expression of these receptors may be regulated by sustained activation of the MAPK p38 and/or ERK. The ESFT cell lines studied differentially expressed the death receptor $p75^{NTR}$, however, there was no correlation between the level of $p75^{NTR}$ expression and induction of cell death by bFGF. Up-regulation of $p75^{NTR}$ occurred 48h after exposure to bFGF in ESFT cells that died following exposure to bFGF, but was unchanged in cells that did not die. To identify whether other apoptotic genes are up-regulated in response to bFGF, a commercially available apoptotic cDNA microarray was probed using total RNA isolated from bFGF-treated and untreated TC32 cells. Three additional death receptors were upregulated in bFGF-treated TC-32 cells compared to the control: CD95/Fas, DR5/KILLER and the TRAIL decoy receptor DcR2. To determine if p38 or ERK signalling is required for up-regulation of death receptors following exposure to bFGF, TC-32 cells were pre-treated with either SB202190 (p38 inhibitor) or PD98059 (ERK inhibitor) and then exposed to bFGF or were left untreated. Western analysis demonstrated that both CD95/Fas and $p75^{NTR}$ are upregulated post-treatment with bFGF when compared to untreated control, and pre-treatment with the p38-inhibitor SB202190 prevented upregulation of these two death receptors. However pre-treatment with the ERK inhibitor PD98059 had no effect on death receptor expression following exposure to bFGF.

### 4.6

THE MITOCHONDRIAL APOPTOTIC PATHWAY IS ACTIVATED BY CISPLATIN IN OSTEOSARCOMA.

[1]Sally Roberts, [2]Tim Eden and [1]Helen Robson* [1]Departments of Clinical Research and [2]Paediatric Oncology, Christie Hospital NHS Trust, Wilmslow Road, Manchester, M20 4BX.

Chemotherapy has dramatically improved the prognosis for osteosarcoma but a proportion of patients still develop drug resistance. Cisplatin (cDDP) is reported to be the most active agent but the mechanisms by which it induces cell death are not clearly defined. Caspases 3 and 9, play an important role in cDDP-induced apoptosis in other cell types. Caspase 9 activation occurs following release of cytochrome c (Cyt c) from mitochondria. We investigated mitochondrial apoptotic pathway activation by cDDP in the HOS cell line.

HOS cells stained with the mitochondrial membrane potential($\Delta\varphi$) sensitive probe JC-1 demonstrated a reduction in within 45 min of cDDP (1-10μg/ml) addition. Cyt c release and pro-caspase 9 and 3 activation were determined by Western blotting. Low levels of Cyt c were found in only the mitochondria of control cells. In cDDP (10μg/ml) treated cells, Cyt c was present in the cytosol and at higher levels in the mitochondria after 1 and 0.5 hours respectively. Pro-caspase 9 was present in cytosolic extracts of control and cDDP treated cells at all time points but was present in the mitochondrial fraction of only cDDP treated cells. These cells showed low levels of cleaved active caspase 9, 0.5 hours after treatment, which significantly increased by 2 hours. High levels of caspase 3 were present in the cytosol of cDDP treated cells at all time points but was virtually undetectable in controls.

These results show activation of a mitochondrial apoptotic pathway by cDDP in human osteosarcoma cells. Greater understanding of the mechanisms involved will assist in efforts to over come resistance.

© 2003 Cancer Research UK

**4.7**

1p DELETED NEUROBLASTOMA CELLS DIFFERENTIATE FOLLOWING CHROMOSOME 1 REINTRODUCTION

Nidal Boulos*, Robert F. Newbold, Deborah A. Trott, Andy DJ Pearson, Mike J.Tilby, John Lunec
*Northern Institute for Cancer Research, Newcastle, NE2 4HH,UK

Aims Chromosome 1p deletions and *MYCN* amplification are common genetic abnormalities in neuroblastoma (NB) correlating with a poor prognosis. Deletion and somatic cell fusion studies suggest that multiple, and as yet unidentified, tumour suppressor genes on 1p are involved in NB development and possibly control *MYCN* expression. Methods & Results This hypothesis was investigated by microcell mediated chromosome transfer. Whole human chromosome 1 (Chr1) transfer into IMR-32 cells (1p deleted and *MYCN* amplified) generated 14 hybrids, 5 of which exhibit morphological changes including neurite outgrowths. The extent of Chr1 incorporated into the hybrids was determined by a panel of 35 Chr1 microsatellite markers. Hybrids carried material from 1q and proximal 1p regions, but none acquired material telomeric to the D1S2890 marker on 1p32.1. Immunofluorescence and western blot analysis showed increased expression of the neurofilament marker NF-200kD in the differentiated hybrids but interestingly, no downregulation of *MYCN* expression. Subsequent transfer of Chr1 fragment (1p36-1q23) into IMR-32 cells failed to generate viable hybrids. To control for procedural effects, Chr17 transfer was performed. Of 26 hybrids, none show altered morphology. Unlike Chr1, whole Chr17 is acquired by IMR-32 cells. Conclusion The results are consistent with the hypothesis that reintroduction of distal 1p (1p36-1p32) is not compatible with growth in NB cells having amplified *MYCN* status. The marked differentiation seen in 5 hybrids and their expression of neuronal markers suggest that other regions of Chr1, particularly 1p21.2-1p21.3, can suppress the transformed status of NB and induce differentiation without altering *MYCN* expression.

**4.8**

FUNCTIONAL ANALYSIS OF TWO *S.pombe* GENES WHICH SHOW A STRUCTURAL SIMILARITY TO THE LEUKAEMIA ASSOCIATED HUMAN *MLLT10* GENE.

Louise K. Jones*[1], Nasser Hajibagheri[2], Vaskar Saha[1], Bryan D. Young[1] and Takashi Toda.[3] Cancer Research UK Children's Cancer Group[1] and Medical Oncology Unit[2], 3rd Floor, John Vane Science Building, Barts and The London School of Medicine and Dentistry, Queen Mary University of London, Charterhouse Square, London, EC1M 6BQ.

Reciprocal chromosomal translocations are associated with acute leukaemias. 5-10% of these translocations result in the fusion of the *MLL* on 11q23 to a number of other genes, including *MLLT10. MLLT10* encodes a 1084aa protein and contains a LAP/PHD zinc finger, together with a functional nuclear localisation signal, AT hook motif and a leucine zipper.

Two AF10 "like" genes have been identified in *S. pombe.* Deletion of both genes PAL1 and PAL2 (*pombe* AF10 Like)indicates that the genes are non-essential in yeast. In addition, Over-expression of human MLLT10 is deleterious for fission yeast. Cells in which MLLT10 is overproduced are highly elongated, reminiscent of cell cycle delay. Nuclear staining with DAPI shows an altered "halo" like chromatin structure, and FACS analysis shows abnormal ploidy. Electron microscopic analysis demonstrates extensive cytosolic vacuolisation and increased numbers of mitochondria and peroxisomes. Immunogold labelling of anti-AF10 antibody localises MLLT10 to the nucleus, and specifically the condensed chromatin. These results may indicate that overproduction of MLLT10 results in the disturbance of pathways involved in chromatin DNA structure and vesicle trafficking by interacting with endogenous yeast proteins.

**5.1**

THE INTERACTION OF THE ETS TRANSCRIPTION FACTOR FAMILY AND ITS CO-REGULATORY PROTEIN AIB-1 PLAYS AN IMPORTANT ROLE IN HER2/ *NEU* OVER EXPRESSING BREAST CANCER

Eddie Myers*, Arnold Hill, Yvonne Buggy, Enda Mc Dermott, Niall O'Higgins, Leonie Young
Department of Surgery, St Vincent's University Hospital and the Conway Institute, UCD, Ireland

HER2/*neu* over expressing breast cancers have a poorer prognosis. Activation of the Ets transcription factors, Ets-1, Ets-2 and PEA3 are implicated in the transcriptional regulation of HER2/ *neu*. The transcriptional activity of Ets is thought to be modulated by nuclear regulatory proteins, including amplified in breast cancer 1 (AIB1).

Our aims were to localise the transcription factors and AIB-1 in breast cancer using immunohistochemistry and to co-localise them using immunofluoresence. The ability of the growth factors bFGF and EGF to modulate the protein expression of the Ets transcription factors in primary breast cultures and breast cancer cell lines was assessed using western blotting.

PEA3, Ets-1, Ets-2 and AIB-1 were localised within breast cancer tissue. The transcription factors and AIB-1 were co-localised to the same cell. Growth factors induced an up-regulation in the protein expression of the Ets transcription factors in a dose dependant manner.

The growth factors, bFGF and EGF regulate Ets transcription factors. Co-localisation with AIB1, implicates nuclear regulatory protein interaction in the modulation of Ets activity in human breast cancer.

**5.2**

LOW RISK PERSISTENT GESTATIONAL TROPHOBLASTIC DISEASE TREATED WITH LOW DOSE METHOTREXATE

Farzana K Khan*, Jan Everard, Samreen Ahmed, Robert E Coleman, Muriel Aitken, Barry W Hancock. Gestational Trophoblastic Disease Centre, Weston Park Hospital, Sheffield

Between 1987 and 2000, 250 patients were treated with intramuscular methotrexate 50mg on alternate days (1,3,5,7) with four doses of oral folinic acid 7.5mg rescue on alternate days (2,4,.6,8), with 7 days between courses.

The overall complete response rate without recurrence was 72% for first line treatment and 95% for those who required second line chemotherapy. Eight women (3.2%) had recurrence following remission and 2(0.8%) had new moles. Two died of their disease giving an overall cure rate of 99%. Only 10 women (4%) experienced grade 3 or 4 toxicity during the first course of treatment and 14 women (5.2%) subsequently. The main toxicities were mucositis and pleuritic chest pain. 141 women (56.4%) became pregnant following treatment; 128 (90.7%) of these were full term normal pregnancies. No second malignancies have been observed.

Methotrexate with folinic acid rescue is an effective treatment for low risk persistent trophoblastic disease. It has minimal severe toxicity, excellent cure rates and does not appear to affect overall fertility. Taking the Charing Cross, London [McNeish et al. J Clin Oncol 2002, 20:1838], and Weston Park, Sheffield, joint experience for all low risk patients treated in the UK (735 over 14 years) the cure rate approaches 100% - a testament to the effectiveness of the national surveillance and treatment scheme.

© 2003 Cancer Research UK

**5.3**

5 YEAR FOLLOW-UP OF A PHASE 3 TRIAL OF NEOADJUVANT CHEMOENDOCRINE THERAPY IN OPERABLE BREAST CANCER Kumar R LAL*, Susan Cleator, Sue E Ashley, Trevor J Powles. *Royal Marsden Hospital, Downs Road, Sutton,, SM2 5PT,*

**Background** We have previously reported a randomized trial of adjuvant versus neoadjuvant chemoendocrine therapy for the treatment of patients with primary operable breast cancer (1) and shown no difference in overall survival (OS) or relapse free survival(RFS). However, those patients in the neoadjuvant arm who responded well to treatment had an improved RFS but not a significantly improved OS compared to those patients who failed to respond.We now update these results with a further 3 years of follow-up data. **Methods** 309 women were randomised to either primary surgery followed by 8 cycles of adjuvant mitoxantrone, methotrexate with tamoxifen (2MT) with or without mitomycin-C(3MT) or the same chemoendocrine regimen for 4 cycles before and 4 cycles after surgery. Radiotherapy was prescribed according to standard criteria. Tamoxifen was continued for 5 years for estrogen receptor positive patients. 142 patients were treated in the adjuvant arm and 144 patients were treated in the neoadjuvant arm. Median age was 56 years (27-70). Patient demographics were balanced between the two arms. Median follow-up of patients is 111 months (range 10 months - 131 months). **Results** There is still no significant difference in OS or RFS when comparing women receiving adjuvant versus neoadjuvant treatment. As in previous reports the 73 patients in the neoadjuvant arm who achieved a complete or near complete response with “minimal residual disease” (CR,MRD) had an improved RFS compared with the 69 patients who achieved a partial response or who demonstrated no change (PR, NC).The 5 year RFS is 87.3% (77.0 - 93.2%, 95%CI) vs 71.4% (58.9 - 80.8%) (p =0.03.). Those neoadjuvant patients who achieved a CR or MRD now have a statistically significant improved OS compared to the patients achieving PR or NR with 89.2%(79.5 - 94.4%) versus 71.4% (59.3 - 80.5%) (p =0.02). **Conclusions** 5 years follow up has shown that clinical response to neoadjuvant chemotherapy is associated with a statistically significant advantage in terms of RFS and OS. To our knowledge this is the first report of an improvement in OS for patients demonstrating a good clinical response that is apparent after prolonged follow-up. 1 MakrisA, Powles T J, Ashley S Eet al. 1998 Ann Oncol 9: 1179.

**5.5**

VITAMIN D IN BREAST CANCER

Kathryn McCarthy*, Christiana A Laban, Sarah E Bonner, Ragheed Al Mufti, Stephen A Bustin, William Ogunkolade, P J Jenkins and R Carpenter.
Depts of Breast Surgery, Barts and The London, Queen Mary School of Medicine and Dentistry.

**Background:** Previous studies have shown an association between vitamin D deficiency and breast cancer risk. Vitamin D is also known to inhibit the mitogenic and anti-apoptotic effects of IGF-1 in breast cancer cells; the bioavailability of IGF-1 is regulated by insulin-like growth factor binding protein-3(IGFBP-3). The local expression of vitamin D in the breast, however, is unknown. Furthermore, it remains to be determined whether these effects involve IGFBP-3.

**Aims**: i)To quantify the expression of 25-hydroxyvitamin D-1-alpha-hydroxylase (1 alpha OHase), the enzyme which converts 25 hydroxyvitamin D to its active metabolite, in paired normal and malignant breast tissue and to correlate this expression with that of IGFBP-3 mRNA and tumour phenotype(histology, oestrogen receptor (ER) status, vascular invasion and lymph node metastases).

**Methods**: Total RNA was extracted from 21 paired samples of normal and malignant breast tissue. Local Ethical Committee Approval was obtained. mRNA transcription was quantified using a real time, hydrolysis probe-dependant RT-PCR assay (‘Taqman’).

**Results**: 1$\alpha$-OHase and IGFBP-3 mRNA were expressed in all samples. 1$\alpha$-OHase was significantly down regulated in normal compared to malignant breast tissue (median copy no $2.27x10^7$ vs $5.62x10^7$, p=0.01). Furthermore, a positive correlation was found between 1 $\alpha$-OHase and IGFBP-3 mRNA expression in the tumours (R=0.61, CI 0.24-0.824, p=0.005). There was no difference in tumour size, grade and ER status between tumours with high and low 1 $\alpha$-OHase expression (+/- $5.00x10^7$).

**Conclusions**: The significant down regulation of 1 $\alpha$-OHase in normal compared to malignant breast tissue supports a role for vitamin D in early breast tumourigenesis. The significant correlation in tumours between 1 $\alpha$-hydroxylase and IGFBP-3 expression supports the view that IGFBP-3 might influence the regulatory effects of vitamin D in breast cancer.

**5.4**

PHASE III STUDY OF VINORELBINE PLUS EPIRUBICIN VERSUS EPIRUBICIN ALONE IN FIRST-LINE CHEMOTHERAPY OF METASTATIC BREAST CANCER: AN SCANDINAVIAN BREAST GROUP STUDY (SBG9403)

Bent Ejlertsen, Henning T. Mouridsen, Sven T. Langkjer, Jorn Andersen, Johanna Sjöström, Mogens Kjaer

The therapeutic benefit of adding vinorelbine to epirubicin was evaluated in patients with metastatic breast cancer.

A total of 387 patients were randomized to receive intravenous vinorelbine 25 mg/m² days 1 and 8 plus epirubicin 90 mg/m² day1 (EV) or epirubicin 90 mg/m² day1 (E). Both study regimens were given every 3 weeks until progressive disease, excessive toxicity or a maximum of one year. Cumulative dose of epirubicin should not exceed 950 to 1000 mg/m². Prior anthracycline-based adjuvant chemotherapy and prior chemotherapy for metastatic breast cancer was not allowed. The primary endpoint was progression-free survival (PFS).

The 2 study arms were well balanced for patient and tumour characteristics. Median number of cycles (9) and median cumulative doses of epirubicin (758 and 729 mg/m²) were similar. Overall response rates (RR) to EV and E were 50% and42%, respectively. The complete RR was significantly higher in the combination arm (17% versus 10%, p =0.048). Median PFS was significantly longer with EV compared to E (10.1 versus 8.2 months, p = 0.019). Addition of vinorelbine to epirubicin reduced the risk of progression by 25% (p= 0.006). Median survival was 19.1 months with the combination and 18 months with epirubicin alone (p = 0.50). Leucopenia-related complications, stomatitis and peripheral neuropathy were more frequent with the combination. Incidences of cardio-toxicity, constipation and injection site reactions were not significantly different between the two arms.

In conclusion, addition of vinorelbine toepirubicin conferred a significant advantage in terms of complete response rate and progression-free survival.

**5.6**

CORRELATION BETWEEN BCL-2 EXPRESSION AND DIFFERENT CLINICO-PATHOLOGICAL VARIABLES IN HUMAN BREAST CANCER

Alaa T Abdel-Rahman*, E Husain, C Wells, and R Carpenter. St Bartholomew’s Hospital, Breast and Endocrine Unit, London.

**Introduction** Bcl-2 plays an important role as an inhibitor of apoptosis that may extend the viability of cells containing genetic alterations and facilitate tumour progression. Inspite of this anti-apoptotic effect, Bcl-2 expression is associated with good prognosis in breast cancer. The aim of this study was to relate Bcl-2 expression to differing clinico-pathological variables in breast cancer.

**Methods** We reviewed fifty-four patients with breast cancer. Immuno-histochemical methods were used together with modified quick score of archival paraffin-embedded sections of tumour. Bcl-2, HER-2/neu, oestrogen receptor, androgen receptor and progesterone receptor were assessed. Expression of Bcl-2 was also related with age, menopausal status, lymph node status, tumour size, tumour grade and tumour types.

**Results** Age range was 29-84 years (mean 59.4Ys). Bcl-2 expression was significantly correlated with increased age >50 years (P-value <0.001), tumour grade I (P-value <0.01), and tumour size <20mm(P-value<0.05). No statistical relations between Bcl-2 expression and menopausal status, lymph node status, tumour types, HER-2/neu expression, oestrogen receptor status, androgen receptor status or progesterone receptor status were found.

**Conclusion** We confirm that Bcl-2 is related to favourable pathological variables. Its assessment in combination with these variables might allow selection of patients with good prognosis who might avoid aggressive systemic therapy.

5.7

DIFFERENTIAL REGULATION OF BREAST CANCER CELL PROLIFERATION BY DIETARY PHYTOESTROGENS.

Jane Limer*, Sarah Burdall, Andrew Hanby, Sally Lane & Valerie Speirs. Molecular Medicine Unit, Clinical Sciences Building, University of Leeds, Beckett Street, LS9 7TF.

The incidence of hormone dependent cancers is lower in Asia as compared to Western nations. The Asian diet is traditionally high in phytoestrogens (PEs), which exhibit putative anti-cancer activities.This research aims to determine the growth effects of the dietary PEs daidzein, genistein and coumestrol in human breast cancer cells, also investigating potential mechanisms of ER-mediated signalling.

The characterisation of ER expression using flow cytometry revealed ER as the predominant receptor subtype in MCF-7 cells, whilst equal levels of ERα and ERβ protein were detected in T47-D. Both ER-positive cell lines demonstrated biphasic growth responses following 0.1-50μM PE treatment, as determined by proliferation assays and cell cycle analysis. Physiological doses of 0.1-10μM genistein and coumestrol stimulated MCF-7 and T47-D growth and S-phase activity, whilst concentrations in excess of 25μM induced $G_2$/M phase arrest. A dose-dependent stimulatory effect of daidzein was observed in MCF-7, however T47-D growth was inhibited only at 25μM. Reporter gene assays suggest the involvement of ER-dependent signalling via the activation of ERE and AP-1 response elements.

We have additionally assessed the effects of PEs on novel primary breast epithelial cultures established from tumour tissue. Tumour cell proliferation was similarly stimulated by PE doses of 0.1-10μM. Our data therefore suggests that the ingestion of dietary PEs may exacerbate the growth of ER-positive breast tumours. Thismay be a cause for concern for post-menopausal subjects using PEs as a natural alternative to HRT.

6.1

TWELVE WEEKS OF NEOADJUVANT CAPECITABINE (CAP) AND OXALIPLATIN (OX) FOLLOWED BY SYNCHRONOUS CHEMORADIATION (CRT) AND TOTAL MESORECTAL EXCISION (TME) IN MRI DEFINED POOR RISK LOCALLY ADVANCED RECTAL CANCER RESULTED IN PROMISING TUMOUR REGRESSION AND RAPID SYMPTOMATIC RELIEF

Ian Chau*, David Cunningham, Diana Tait, Gina Brown, Niall Tebbutt, Mark Hill, Andrew Wotherspoon, Andrew Norman, Alison Massey *Department of Medicine, Royal Marsden Hospital, Downs Road, Sutton, Surrey SM2 5PT

**Purpose:** To evaluate neoadjuvant cap/ox prior to CRT and TME in newly diagnosed patients (pts) with MRI defined poor risk/locally advanced rectal cancer. **Methods**: Pts received 12 weeks of neoadjuvant cap (2000mg/m$^2$/day po for 14 days every 3 weeks) and ox (130mg/m$^2$ iv every 3 weeks). Starting on week 13, cap was continued at 1650mg/m$^2$/day continuously with concomitant radiotherapy. TME was planned 6 weeks after CRT. Post-operatively, pts received 12 weeks of cap at 2500mg/m$^2$/day. MRI was repeated after chemotherapy and CRT. **Results**: Between November 01 and November 02, 22 eligible pts were recruited. Median age was 62 (range=38-80). So far, 17 patients are evaluable for radiological response and 15 patients have proceeded to TME. Following neoadjuvant cap/ox, all pts had partial responses (RECIST criteria). In addition, 80% of pts had symptomatic responses in a median of 22 days (i.e. after one cycle of chemotherapy) including reduced rectal bleeding (100%), improvement in diarrhoea/constipation (79%), diminished pelvic pain/tenesmus (64%) and weight gain/stabilisation (100%). Following CRT, tumour response was sustained in all pts. Of 20 pts with initial threatened circumferential resection margin (CRM), tumour regression away from the CRM occurred in all pts who have completed treatment so far allowing R0 resection in 100% of these pts. Pathological CR was found in 5 pts. **Conclusion**: Capecitabine and oxaliplatin prior to synchronous CRT and TME produces almost universal tumour regression, rapid symptomatic response and allows R0 resection to be achieved.

5.8

FACTORS PREDICTING AXILLARY LYMPH NODE INVOLVEMENT IN PATIENTS WITH SMALL BREAST CANCERS

*Sankha.S.Mitra ,Sue Ashley, Aseem.Y.Rostom
Correspondence address: 41 Alexandra Avenue,Sutton,Surrey SM1 2PA

We carried out a retrospective analysis of patients with tumours <10mm seen at the Royal Marsden Hospital between 1990 and 2002 from our perspective database. All patients had axillary node dissection and we have full histological information on lymphovascular invasion, ER/PR status, number of involved nodes and the tumour grade. Patients who received neoadjuvant chemo/endocrine therapy or preoperative irradiation were excluded.

Results: 452 patients met the analysis criteria.

Discussion: Ninety-eight out of the 452 patients (21.7%)with tumours under 10mm had axillary lymph node involvement. Even in grade I tumours, there is a 12.8% incidence of nodal involvement. With holding axillary sampling in these patients may result in understaging of a significant number of patients.

We found vascular invasion and grade of the tumour to be independent predictors for nodal involvement. 58% of the node +ve patients had vascular invasion while only 41%of the node +ve patients had no vascular invasion. The incidence of nodal involvement increased with grade, being 12.8% for grade I tumoursand 29% for grade III tumours. Higher grade tumours tended to have a higher incidence of vascular invasion.

We found that 56% of the node +ve patients were ER +ve while only 15.3% of node +ve patients were ER-ve. This is thought to reflect the higher frequency of ER +ve patients (276/452) skewing the data as 19.9% of ER +ve patients were node +ve compared with 26.7% of ER-ve patients. Therefore, a higher percentage of ER-ve patients seem to have nodal involvement.

Conclusion: Vascular invasion, grade and ER –ve status are all predictive factors for lymphnode metastasis.

6.2

LATE EFFECTS FOLLOWING TOTAL MESORECTAL EXCISION AND SHORT COURSE PREOPERATIVE RADIOTHERAPY

Margaret King[1*] Shaun Tolan[1], Selveraj Giridharan [1], Chris McConkey[2], Andrew Hartley[1], Ian Geh[1]
[1]Cancer Centre, Queen Elizabeth Hospital, Birmingham B15 2TH
[2]Cancer Research UK Clinical Trials Unit, University of Birmingham

**Introduction** Late side-effects following short course preoperative radiotherapy (SCPRT) and total mesorectal excision (TME) for operable rectal cancer have not been adequately studied. Acute toxicity in a series of 176 consecutive patients has been previously reported. In this study, late effects in the same cohort are presented.

**Method** Side effects occurring more than 3 months after the start of SCPRT were graded using the EORTC/RTOG late radiation toxicity system. Multivariate analysis was performed to identify associated factors

**Results** Of 176 patients, 15 died within three months of SCPRT and 5 patients were lost to follow up. At a median follow up of 40 months severe (grade 3-4) toxicity was seen in 20 (13%) of 156 assessable patients: Gastrointestinal 13 (8%); Other 7 (5%). On multivariate analysis, abdominoperineal (AP) resection (p<0.001) was associated with less severe toxicity.

**Conclusions** In this retrospective series the rate of late grade 3-4 toxicity following SCPRT and TME was 13%. Although AP resection is associated with a lower incidence of late side-effects this may be counterbalanced by the impact of a stoma on quality of life. These factors should be considered when deciding on the optimal management of operable rectal cancers.

© 2003 Cancer Research UK

### 6.3

CHEMORADIATION IN LOCALLY ADVANCED RECTAL CANCER USING BOLUS 5-FLUOROURACIL, Suzannah Mawdsley*[1] & Rob Glynne-Jones. [1]The Gray Cancer Institute, Mount Vernon Hospital, Northwood, Middlesex HA6 2RN, UK.

**Introduction**: Chemoradiation prior to surgery is now a common approach in the treatment of locally advanced rectal cancer. This report describes the resection, survival and local relapse rates of patients treated over an 8-year period at Mount Vernon Hospital.

**Methods**: Between 1994 and 2002, 155 patients with a locally advanced adenocarcinoma of the rectum were treated. T stages were: T2:2, T3:59, T4:94. Median age 67 years, male: female ratio 73:27%. The radiotherapy dose was 45Gy in 25 fractions over 5 weeks. 5-FU was given as a 60 minutes infusion on days 1-5 and 29-33, at 350mg/m$^2$, following low dose folinic acid. Surgery was performed in 114 patients. Actuarial survival, local control and toxicity rates according to CTC criteria, were assessed.

**Results**: 93% of patients achieved 100% compliance during chemoradiation, common toxicities were diarrhoea, tenesmus and skin reactions. Resected specimens revealed 12% had a complete pathological response (CPR), downstaging occurred in 41%, in 37% there was no change and 10% were unresectable. R0 resections were achieved in 95/155 (61%). Overall median survival was 37 months with a 5-year survival of 34%. Median time to local recurrence (LR) was 22 months and occurred in 8% of R0 and 11% of R1/2 resections. 54/155 (35%) cases developed distant metastases in a median time of 19 months.

**Conclusion**: Chemoradiation with 5-FU can produce effective downstaging in patients with locally advanced rectal carcinoma and toxicity is manageable. Survival appears poor in this group and the results of the Phase III EORTC trial with 5FU/folinic acid are awaited. Phase two studies are in progress to investigate the use of newer agents in combination with 5FU, which may not only improve LR and R0 resection rates but also impact upon survival.

### 6.4

LYMPH NODE STAGING FOLLOWING PREOPERATIVE CHEMORADIATION IN RECTAL CANCER. Mark Beresford*, Robert Glynne-Jones, Andreas Makris, Mark Harrison. Mount Vernon Cancer Centre, Northwood, Middlesex HA6 2RN.

**Introduction:** The lymph node (LN) status of rectal cancers is a key prognostic indicator. UICC guidance suggests that at least 12 LNs should be examined before a tumour can be pronounced node-negative. Others suggest that 7 or 8 LNs might be sufficient, but with increasing usage of neoadjuvant chemoradiation (NCRT) the effacement of LNs means that often far fewer are found.

**Patients and methods**: 185 patients with T3 or T4 rectal cancer treated with NCRT between 1994 and 2002 have been entered into a prospective database. All patients were deemed inoperable on initial staging. Treatment was with concurrent radiotherapy (45 Gy in 25 fractions over 5 weeks) and leucovorin/5FU chemotherapy.

**Results**: 134 males and 51 females had a mean age of 63. A total of 161 patients (pts) proceeded to surgery. The mean number of LNs found in the resection specimens was 5.47 (range 0-21). Complete pathological response was seen in 19 pts (12%). Overall 3-year survival figures were 54%. For node positive pts this fell to 43%, with a disease free survival (DFS) of 16%. In node negative pts with 2 or less nodes examined, 3-year survival figures were 47% (DFS = 26%). Where 3 or more negative nodes were found, overall survival was 66% (DFS = 58%).

**Conclusions:** LN status following chemoradiation is predictive of survival. There is considerable effacement of LNs, resulting in a yield lower than would be traditionally required for prognostic information. We demonstrate that if more than 2 negative nodes are found, the outcome is more favourable than if 2 or less negative nodes are identified. It seems likely that this is due to understaging of pts in whom insufficient nodes are recovered, and we suggest that following NCRT at least 3 nodes are required before a specimen can be considered truly N0.

### 6.5

THE MRC CLASICC TRIAL: RESULTS OF SHORT-TERM ENDPOINTS

Pierre J. Guillou*, Phil Quirke, Nick Bosanquet, Adrian Smith, Helen Thorpe, Joanne Walker, Sue E. Bell, Julia M. Brown. Northern & Yorkshire Clinical Trials & Research Unit, University of Leeds, UK.

This large prospective, multicentre, multidisciplinary, pragmatic, phase III trial was designed to compare conventional (Open) versus laparoscopic-assisted (Lap) surgery in colorectal cancer in terms of pathological, clinical and health economic outcomes. Patients were randomised in an unbalanced allocation of 2:1 Lap: Open resection. Recruitment closed on 31 July 2002 with a total of 794 patients (526 Lap, 268 Open).

Primary short-term endpoints were pathological resection margins and 30-day operative mortality. Secondary short-term endpoints were blood transfusion requirements, operative complication rates and short-term quality of life. All pathological data were centrally reviewed. Longer-term endpoints are defined as recurrence and metastases, disease-free and overall survival.

Pre-treatment characteristics: 413 colon, 381 rectal, median age 70 years, 56% male, 87% WHO performance status 0-1. Data were analysed in an intention-to-treat analysis. Circumferential margin positivity rate was 13% (Open) and 15% (Lap), $p=0.68$; longitudinal margin rate was 0% (Open) and 1% (Lap), $p=0.31$; 30-day operative mortality was 4% in both arms, $p=0.82$; 30-day complication rate was 36% in both arms, $p=0.97$. Pathological data were excellent with lymph node median yield of 13 overall.

This trial provides an early indication of the role of laparoscopic resection in colorectal cancer. The short-term results suggest that, in the UK, the laparoscopic-assisted technique for colorectal cancer appears to be no different from open surgery. It is the only trial considering rectal cancer, and is unique in its high-quality pathological data collection and pathological endpoints.

### 6.6

THE DEVELOPMENT OF α-FOLATE RECEPTOR (α-FR) TARGETED THYMIDYLATE SYNTHASE (TS) INHIBITORS

Ann L. Jackman*, David Gibbs, Davinder Theti, Matthew Green, Florence Raynaud, Melanie Valenti, Vassilios Bavetsias. Haddow Laboratories, Institute of Cancer Research, Sutton, Surrey.

The targeting of anticancer drugs to tumours seeks to exploit a difference in the biology of tumours compared with that of normal tissues. One such difference is the high expression of the α-FR in some epithelial tumours e.g. 90% of ovarian. The α-FR, linked to increased susceptibility to malignant transformation and regulation of tumour proliferation, also is a high affinity, low capacity transporter of folates. The reduced-folate carrier (RFC) is the primary folate/antifolate transporter and its ubiquitous expression leads to antifolate cytotoxic effects in tumours and normal tissues. TS inhibitors such as raltitrexed, pemetrexed and ZD9331 are also co-transported via the α-FR in some preclinical models. Thus, we hypothesised that a TS inhibitor displaying high and low affinity or the α -FR and RFC respectively could be an α-FR-targeted drug. Two cyclopenta[*g*]quinazoline-based compounds, CB300638 and CB300945, possess this profile (TS $K_i$ = 0.24nM and 1.4nM respectively). Following rapid binding to the α-FR they are slowly trafficked via an endosomal pathway. The 72h $IC_{50}$ in A431-FBP (α-FR transfected) and KB (α-FR constitutive) cells is ~3nM while in α-FR negative lines is ~1 to 10μM. Both compounds localised to the KB xenograft in mice (100mg/kg i.p. bolus) compared to normal tissues. Target inhibition in tumour, but not in normal tissues, was demonstrated using $^{125}$IdUrd metabolism as a surrogate marker of TS inhibition. By contrast, selective targeting in this model was not seen with ZD9331. CB300638 and CB300945 have been selected as potential drug candidates that are predicted to display efficacy in α-FR$^+$ tumours and very low toxicity. *Supported by Cancer Research UK.*

© 2003 Cancer Research UK

### 6.7

LOCAL IMMUNE RESPONSE IN ANAL HPV RELATED LESIONS. COMPARISON BETWEEN HIV POSITIVE AND HIV NEGATIVE POPULATIONS

*J. Mullerat[1], F. Deroide[2], B. Lloveras[3], W. Quint[4], M. C. Winslet[1], M. Bofill[5]

University Department of [1]Surgery, [2]Histopathology, [5]Immunology Royal Free and University College Medical School, London, U.K, [3]HPV Laboratory, Institut Catala d'Oncologia, L'Hospitalet, Barcelona, Spain, and [4]Delft Diagnostic Laboratory, The Netherlands.

Most cases of anal carcinoma develop from high grade anal intraepithelial neoplasia (AIN) caused by persistent human papillomavirus (HPV) infection. We studied the host's local cellular immune response in HPV-related anal lesions and compared the results between HIV + and HIV - groups. This study assessed the degree of local T lymphocytic infiltrate (CD-3, CD-4 and CD-8) in the stroma and epithelium of HPV related anal lesions in 75 patients (41 HIV – and 34 HIV+) with immunohistochemistry, and the HPV serotype/s by PCR detection of carcinogenic HPV types (16, 18, 31, 33, 35, 39, 45, 51, 52, 56, 58, 59 and 68).

There was a local lymphocytic infiltrate in the stroma and epithelium, in all groups of lesions. The T cell infiltrate (CD4 and CD8) showed a significant ($p<0.05$) increase as the lesions became gradually dysplastic and invasive both in HIV – and HIV + groups. The cell infiltrate was significantly lower in the HIV + group for each group of lesions, mostly at the expense of CD4 cells. 74% of lesions in the HIV – group and 88% in the HIV + group contained carcinogenic HPV types. This difference was not statistically significant.

This is the first study that shows a local cellular immune response in anal HPV lesions. These data also suggest that the poorer prognosis in HIV + patients is likely to be due to their inherent cellular immunosupression, rather than a higher exposure to carcinogenic HPV types.

### 7.1

PATIENT'S EXPERIENCES OF LUNG CANCER DIAGNOSIS

Meinir Krishnasamy & Erna D Wilkie*
Correspondence Address: Erna D Wilkie. Research Nurse, Department of Cancer Medicine, Ninewells Hospital & Medical School, Dundee, DD1 9SY, Scotland.

This paper draws on data drawn from two studies undertaken to assess the health care needs of patients with primary lung cancer. In the first of the two studies (a national mail questionnaire survey), 209 (45%) patients accessed from 24 randomly selected hospitals throughout the UK, returned completed questionnaires. Participants responded to questions relating to their experiences of care provision from time of first presenting to their GP, on through treatment and into follow-up. The second of the two studies, draws on in-depth interview data gathered from a cohort of 60 patients with lung cancer who agreed to take part in interviews at one, three and six months post diagnosis.

Although the considerable impact of the diagnostic process and of its "telling" on cancer patients' construction of their experience of care are well documented, little is known of the process and associated outcomes of the diagnostic pathway for patients with lung cancer. Given that the median survival for patients with lung cancer after diagnosis is less than four months, with about 80% of patients dying within one year[1], the need to ensure that the diagnostic process is swift, well co-ordinated and therapeutic in its own right, is imperative.

Data from our studies suggest that work needs to be undertaken, and its effect evaluated, to better inform the public of symptoms that warrant urgent attention.

### 6.8

RANDOMISED, MULTICENTER PHASE III STUDY COMPARING CAPECITABINE WITH FLUOROURACIL AND OXALIPLATIN WITH CISPLATIN IN PATIENTS WITH ADVANCED OESOPHAGOGASTRIC CANCER: CONFIRMATION OF DOSE ESCALATION.

Catherine Harper-Wynne*, Kate Sumpter, David Cunningham Andrew Norman, Jaqui Oates, Claire Durrant,Tim Iveson, Marianne Nicholson, Tamas Hickish, Mark Hill,.*Royal Marsden Hospital, Downs Rd, Sutton Surrey SM2 5PT.

PURPOSE: To establish the potential use of the third generation platinum compound, oxaliplatin (O) & the oral fluoropyrimidine capecitabine (X) in untreated patients (pts) with advanced oeophagogastric cancer, using a multicenter randomised study.
METHODS: Pts were randomised to 1 of the 4 regimens; epirubicin, cisplatin, fluorouracil (ECF), EOF, ECX or EOX. Doses: E 50mg/m$^2$, C 60mg/m$^2$ & O 130mg/m$^2$ IV 3 weekly; F 200mg/m$^2$/day IV & X 1000mg/m$^2$/day (escalated to 1250mg/m$^2$/day) PO, continuously. The first interim analysis was performed after 80 pts had been randomised. 5.1% of X treated pts experienced grade 3/4 toxicity of the dose limiting fluoropyrimidine toxicities; stomatitis, palmar plantar erythema (PPE) and diarrhoea. A protocol dose escalation to 1250mg/m$^2$ was therefore instituted and a 2$^{nd}$ interim analysis has been performed.
RESULTS: 204 pts have been randomised. From 176 pts evaluable for toxicity 10.7% of the 1250mg/m$^2$ capecitabine group experienced non-haematological grade 3/4 toxicity. From 153 pts evaluable for response, combined CR + PR rates for each regimen are: ECF 31%, EOF 33%, ECX 35% and EOX 52%.
CONCLUSIONS: The non-haematological toxicity of 10.7% in the 1250mg/m$^2$ capecitabine group confirmed this as the optimal dose, which will therefore be used until the planned total accrual of 600 patients. Updated data will be presented at the meeting.

### 7.2

THE USE OF MVP CHEMOTHERAPY IN MALIGNANT MESOTHELIOMA: OUTCOME AND PREDICTIVE FACTORS

E Andreopoulou*, PJ Ross, MER O'Brien, A Norton, K Priest, S Ashley, S Popat, C Harper-Wynne, K Sumpter, IE Smith. Lung Unit, Royal Marsden Hospital, Downs Road, Sutton, Surrey, SM2 5PT.

This study aimed to assess the palliative benefit of MVP chemotherapy in patients (pts) with mesothelioma and assess prognostic factors. Pts with histologically confirmed mesothelioma were treated with mitomycin-C 8mg/m$^2$ (courses 1,2,4 &6), vinblastine 6mg/m$^2$ (maximum 10mg) and cisplatin 50mg/m$^2$ IV q 21 days for up to 6 cycles. 150 pts were treated and analysed for objective and symptomatic tumour response, toxicity and survival. The overall response rate was 15.3% (95% CI 9.3–21.4%) and 68.6% demonstrated no objective change. 147 pts reported symptoms at the start of chemotherapy and 69% had a symptomatic response to MVP, including 2 pts with a complete resolution of all symptoms. There were no toxic deaths. The most frequent grade 3/4 non-haematological toxicities were infection (12%), nausea/vomiting (9%) and constipation (5%). Grade 3/4 haematological toxicities were neutropaenia (22%), anaemia (6%) and thrombocytopaenia (5%). Median survival was 7 months and at 1-year 31.6% (95% CI:24.0–39.5%) were alive. Univariate analyses demonstrated that poor performance status (WHO 2/3) ($p=0.003$), weight loss ($p=0.009$), non-epithelioid histology ($p=0.003$), low haemoglobin (<13 g/dl in males and <11.5g/dl in females) ($p=0.008$), low white cell count ($<11\times10^9$/l) ($p=0.008$) and low absolute neutrophil count ($<5.0\times10^9$/l)($p=0.02$) were all significant prognostic factors. Multivariate analysis demonstrated that weight loss (relative risk [RR] 3.34,p=0.001), non-epithelioid histology (RR 2.69,p=0.01), low haemoglobin (RR 2.41, p=0.01), low white cell count (RR 2.94,p=0.001) were all independent prognostic factors.
We conclude that MVP is a palliative regimen which can be given safely even to pts with poor performance status. In addition, we have confirmed that weight loss, non-epithelioid pathology, and anaemia are poor prognostic factors. To improve outcomes treatment strategies will need to address symptoms such as weight loss and anaemia.
Dr E Andreopoulou was supported by an ESMO Fellowship

© 2003 Cancer Research UK

### 7.3

CHART TO CHARTWEL TO CHEMOTHERAPY AND CHARTWEL IN NON-SMALL CELL LUNG CANCER

B E Lyn*, E M Wilson, F J Williams, M I Saunders, H Phillips
Mount Vernon Cancer Centre, Mount Vernon Hospital, Northwood, Middlesex, HA6 2RN, UK

The CHART trial showed a 9% improvement in survival, 29% compared with 20%, in patients with locally advanced inoperable non-small cell lung cancer treated with CHART compared with conventional daily radiotherapy to 60Gy. As centres found treatment on week ends difficult, the CHARTWEL, CHART Week End Less, protocol was developed and in a feasibility study the dose was gradually increased to 60Gy in 17 1/3 days. Three courses of neo-adjuvant chemotherapy were then added prior to CHARTWEL. Initially, chemotherapy was with the MIC (Mitomycin, Ifosfamide and Cisplatin) regime but later PC (Paclitaxel and Carboplatin) was used. Other regimes included MVP (Mitomycin, Vinblastine and Cisplatin) and VC (Vinorelbine and Cisplatin).

The 2 year survival was 40% in 20 patients treated to <60Gy, 47% in 55 patients treated to 60Gy and 47% in 33 patients treated with chemotherapy and radiotherapy. The addition of chemotherapy led to increased dysphagia but reactions settled in all cases. Twenty four percent of those receiving chemotherapy and radiotherapy suffered grade 2 or worse lung morbidity, higher than those treated with radiotherapy alone, but there was no significant difference between the schedules. There were no cases of myelopathy.

Induction chemotherapy followed by CHARTWEL to 60Gy is feasible and there may be a therapeutic benefit.

### 7.4

ADDITION OF SRL172 TO STANDARD CHEMOTHERAPY IN SMALL CELL LUNG CANCER (SCLC) CONFERS NO SURVIVAL BENEFIT: RESULTS OF A RANDOMISED MULTICENTRE STUDY

K. Sumpter*, C.L. Harper-Wynne, M.E.R. O'Brien, P. Johnson, R.Bass, T. Goldie, A. Norton, P. Ross, H. Ford. Royal Marsden Hospital, Downs Rd, Sutton; Kent Cancer Centre & CRUK Oncology Unit, Southampton.

Background: SRL172 is a suspension of heat killed *Mycobacterium vaccae,* found to be a potent immunological adjuvant when used with autologous cells in vivo. A previous phase II study in SCLC revealed a trend to improved median survival with the combination of chemotherapy and SRL172 and no increased toxicity irrespective of drug regimen used [Assersohn et al 2001]. We report a randomised multicentre study assessing the efficacy and safety of standard chemotherapy with or without SRL172 in SCLC.

Methods: Patients had histologically confirmed limited or extensive SCLC and WHO performance status ≤ 2. Patients could receive either an anthracycline or platinum based regimen with randomisation to receive intradermal SRL172 at day 0 prior to chemotherapy and weeks 4,8,12, and 16. On completion of chemotherapy SRL172 was continued every 8-12 weeks and on progression SRL172 was continued if further treatment of any kind was considered. All patients were followed for survival.

Results: 76 patients were randomised and 72 received carboplatin and etoposide chemotherapy. There was no difference in response rates between the two arms, 73% without vaccine and 68% with vaccine. Median duration of response and median survival was 9 months for both groups. The standard arm (chemotherapy only) response rate and survival was better than the pilot study.

Conclusion: Despite encouraging phase II results, this study suggests no advantage to response or survival in adding SRL172 to standard treatment in SCLC. Improved results in the standard arm of this phase III study compared to the anthracycline/low dose platinum regimen used in the phase II study may account for the lack of difference. Putting together this result with SRL172 in SCLC and the results of SRL172 in NSCLC, the strategy of combining SRL172 with chemotherapy will not be pursued. This study was supported by SR Pharma.

### 7.5

SMALL CELL LUNG CANCER: CAN PROGNOSTIC MODELS HELP US IN THE CLINIC?

Ewan Brown*, Melanie Mackean
Edinburgh Cancer Centre, Crewe Road South, Edinburgh, UK.

**Background:** Prognostic models have been extensively studied in small cell lung cancer. The 'prognostic tree model' (Sagman et al. J Clin Oncol 9:1639-1649,1991) divides patients into four distinct groups according to routinely collected clinical and laboratory data. This retrospective review aims to assess whether this model divides patients into therapeutically important groups when applied to a cohort of patients with small cell lung cancer in South-East Scotland.

**Methods:** Between 1999-2000, 162 patients with small cell lung cancer were identified through the cancer registry at the Edinburgh Cancer Centre and variables including age, performance status, extent of disease and laboratory parameters (including LDH, WCC and AlkPhos) were assessed and survival for each prognostic group was calculated.

**Results:** Patients had a median age of 67 (range 38-88). 69 patients had limited disease (43%). Median survival was 89 days for extensive disease patients and 415 days for limited disease. When divided into prognostic groups according to the 'tree model' the median survivals for the 4 groups were 484 days for group A, 293 days for group B, 120 days for group C and 46.5 days for Group D respectively.

**Conclusion:** The 'tree model' divides patients into 4 easily definable groups when applied to this cohort of patients. Use of the model provides useful prognostic information and may help in the clinic in targeting patients for specific treatments. In particular it may be useful in selecting patients within the limited or extensive disease groups who may benefit from more aggressive therapy and those who will not.

### 7.6

DETECTION OF RARE DISSEMINATED TUMOUR CELLS IDENTIFIES HEAD AND NECK CANCER PATIENTS AT RISK OF TREATMENT FAILURE

Max Partridge*, Ruud Brakenhoff, Elaine Phillips, Kulsan Ali, Rebecca Francis Richard Hooper, Kenneth Lavery, Andrew Brown, John Langdon. Correspondence address Maxillofacial Unit/Oncology, King's College Hospital, London, SE5 8RX, UK.

In this study, samples of bone marrow (BM) and venous blood collected preoperatively and three months post-surgery for 40 head and neck cancer patients, were screened for the presence of disseminated tumour cells (DTCs) using immuncytochemistry (ICC) with a pan cytokeratin antibody and *E48* RT-PCR to establish the most appropriate samples and assay to incorporate into prospective studies. The molecular approach was also applied to intraoperative central venous blood (CVB). the concordance between the molecular and ICC tests applied to preoperative samples, measured by Cohen's kappa, was not uniformly good (BM 0.62, CVB 0.12), likely reflecting sampling errors, heterogeneity of E48 antigen expression or stochastic effects. However, testing samples of BM and CVB preoperatively with the molecular or ICC approaches gave results that predicted disease free survival (DFS) and distant- metastases free survival (DMFS). Application of a single preoperative test predicted development of distant metastases but there was also evidence that the prediction could be improved by combining information derived from testing both CVB and BM. Testing the intraoperative sample of CVB was also a sensitive predictor of distant metastases, but testing BM or peripheral venous blood three months post-surgery was not useful. The discordance between the results obtained with the postoperative *E48* RT-PCR and ICC may reflect the presence of epithelial cell debris in the haematopoietic cell compartment (HCC) after radiotherapy. These findings suggest that detection of DTCs pre- or intraoperatively signals for a high risk of local and distant recurrence and reduced survival in head and neck cancer.

**7.7**

OUTPATIENT CHEMO-RADIOTHERAPY FOR LOCALLY ADVANCED HEAD AND NECK CANCER USING WEEKLY CISPLATIN Gwo F Ho*, Caroline H Bridgewater, Melanie EB Powell, Dept of Radiotherapy,
St. Bartholomew's Hospital, London EC1A 7BE

**Aim**: To determine the feasibility and toxicity of a cisplatin based outpatient chemo-radiation regimen in locally advanced squamous cell carcinoma (SCC) of the head and neck.

**Methods**: Patients with locally advanced head and neck cancer, performance status 2 and GFR 50ml/min were considered for radical chemo-radiation. Radiotherapy (66Gy in 33 daily fractions) was given with concurrent weekly outpatient cisplatin (40mg/m$^2$) and acute toxicity assessed weekly. Initial clinical response was evaluated at 3 months post treatment.

**Results**: Since May 2000, twenty patients have received this schedule. A mean number of 4.7 chemotherapy cycles were given (range 2-6) and the median duration of radiotherapy was 44.5 days (range 37-47). All patients completed radiotherapy as planned. Six patients required a total of 9 hospital admissions for treatment related toxicity. Causes were nausea/vomiting (4), neutropaenic sepsis (2), constipation (2) and mucositis (1). Eight patients required blood transfusion and 2 had asymptomatic grade 3 neutropenia. One developed total dysphagia. Skin and mucosal reactions were not unduly severe, and had healed in all patients by 12 weeks, one has subsequently developed radiation necrosis. With a median follow up of 5 months (range 1-19), there have been 16 complete responses, 2 partial responses, and 2 patients with progressive disease. Two patents have died with local recurrence.

**Conclusion**: Chemoradiotherapy with weekly outpatient cisplatin is a well tolerated and feasible treatment for advanced head and neck SCC. Response is encouraging but long term results are awaited.

**7.8**

INFORMATION NEEDS OF ASIAN AND WHITE ENGLISH CANCER PATIENTS AND THEIR FAMILIES IN LEICESTERSHIRE: A CROSS SECTIONAL SURVEY

R Paul Symonds*, D Muthu Kumar, Santhanam Sundar, Kausher Ibrahim, Boki SP Savelyich, Ed Miller Department of Oncology and University of Leicester, Leicester Royal Infirmary Leicester LE1 5WW

The aim of the study was to find the information needs of British Asian cancer patients. An additional objective was to find the extent of family involvement when the patient was given the cancer diagnosis and the patient's views about information disclosure. 82 Asian patients and 220 random white English control patients visiting the Leicestershire cancer centre were included in a questionnaire survey. This is the first survey of the information needs of an ethnic minority group with cancer.

More white English patients gave positive answers to the statement "I want as much information as possible" than Asian patients (93.1% v 77.5% p =<0.001). However, 92.6% of Asian patients wanted to know if they had cancer. Many more Asians (66.2% v 51 % p < 0.001) indicated their GP was the preferred source of information. The vast majority of both Asian and English patients agreed that family or friends should be present when patients are given the cancer diagnosis. However, Asians were more likely to be alone 24% v 15% (p = < 0.008) when told they had cancer. The majority of patients (both white English and Asian) want to control the disclosure of information to relatives and friends and would like to be present at doctor/family meetings. We suggest all patients attending an appointment where they will be told bad news should be invited to bring friends or family members. Further research is needed to find how to increase the role of the GP in giving diagnostic and prognostic information to Asian patients.

© 2003 Cancer Research UK

